# Transcriptomic profiling of induced steatosis in human and mouse precision-cut liver slices

**DOI:** 10.1038/s41597-023-02220-0

**Published:** 2023-05-19

**Authors:** Eric Simon, Maciej Motyka, Grietje H. Prins, Mei Li, Werner Rust, Stefan Kauschke, Coralie Viollet, Peter Olinga, Anouk Oldenburger

**Affiliations:** 1 Global Computational Biology and Digital Science, Research Boehringer Ingelheim Pharma GmbH & Co.KG, Biberach, Germany; 2 Ardigen, Podole 76, 30-394 Kraków, Poland; 3 Department of Pharmaceutical Technology and Biopharmacy, University of Groningen, Groningen Research Institute of Pharmacy, A, Deusinglaan 1, 9713AV Groningen, The Netherlands; 4 CardioMetabolic Diseases Research, Boehringer Ingelheim Pharma GmbH & Co.KG, Biberach, Germany

**Keywords:** Metabolic syndrome, Computational biology and bioinformatics

## Abstract

There is a high need for predictive human *ex vivo* models for non-alcoholic fatty liver disease (NAFLD). About a decade ago, precision-cut liver slices (PCLSs) have been established as an *ex vivo* assay for humans and other organisms. In the present study, we use transcriptomics by RNASeq to profile a new human and mouse PCLSs based assay for steatosis in NAFLD. Steatosis as quantified by an increase of triglycerides after 48 h in culture, is induced by incremental supplementation of sugars (glucose and fructose), insulin, and fatty acids (palmitate, oleate). We mirrored the experimental design for human vs. mouse liver organ derived PCLSs and profiled each organ at eight different nutrient conditions after 24 h and 48 h time in culture. Thus, the provided data allows a comprehensive analysis of the donor, species, time, and nutrient factor specific regulation of gene expression in steatosis, despite the heterogeneity of the human tissue samples. Exemplified this is demonstrated by ranking homologous gene pairs by convergent or divergent expression pattern across nutrient conditions.

## Background & Summary

Excessive caloric intake causes liver steatosis and can lead to non-alcoholic fatty liver disease (NAFLD)^1,2^. With persistent nutrition overload, NAFLD can further progress to non-alcoholic steatohepatitis (NASH) which is characterized by liver inflammation and liver fibrosis at later stages that can ultimately lead to liver cirrhosis and failure^3^. Although NASH is going to become a major risk factor for these hard endpoints, there are no registered drugs for treatment available yet^4^. A major hurdle for new therapies is the complexity of the disease because it involves multiple cell types and molecular processes that are highly interconnected. In particular, there is a gap between cellular *in vitro* models and subchronic *in vivo* models that hinders the translation of preclinical findings into the clinic^5^.

Precision-cut liver slices (PCLSs) from human tissues have been established as a major *ex vivo* model for preclinical drug testing^6,7^. Accordingly, PCLSs represent a robust and reliable assay which can be scaled up to complex conditions with multiple experimental factors with a small amount of liver material and a small number of liver organ samples. The molecular processes which are induced within the first 48 h in PCLS culture have been extensively characterized previously^8^. We have now optimized PCLSs to mimic metabolic conditions of nutrition overload and thus recapitulate major hallmarks of NAFLD *ex vivo* by incrementally adding sugars, insulin and fatty acids to the PCLSs media which ultimately leads to steatosis as quantified by triglyceride quantitation and inflammation as measured by cytokine release. The present study provides the full transcriptomic features of this new disease-relevant NAFLD assay.

To better understand the intra- and inter-donor variability of the essential assay readouts (i.e. triglyceride synthesis and cytokine release), we have designed a perfectly balanced experiment using liver organ samples from seven human donors. From each liver, we sequenced PCLSs after 24 h and 48 h in culture at baseline conditions and with incremental supplementation of glucose, fructose, insulin, palmitate, and oleate to the PCLS culture. In a second experiment, we applied the same setup using PCLSs from five healthy male C57/BL6 mice in order to compare the human data to the data from the controlled animal experiment. An overview of the donated human samples is given in Table 1. The experimental design is given in Table 2.

**Table 1:** Overview of human sample donors.

Donor	Procedure (excision date <M-YYYY>)	Cold Ischemia Time (h)	Baseline TG (µg/mg)
H1	Resized transplantation liver (10-2016)	5	23.5
H26	Donation after cardiac death (10-2020)	29	11.0
H27	Hemihepatectomy (12-2020)	5	41.9
H29	Hemihepatectomy (12-2020)	5	6.1
TX06	Resized transplantation liver (3-2018)	3	40.6
TX08	Hemihepatectomy (5-2018)	28	40.1
TX25	Resized transplantation liver (2-2018)	3	42.3

Human liver biopsies that were used to prepare the PCLSs with total cold ischemia time from organ procurement to tissue culture and the baseline triglyceride levels. For each condition, three PCLSs from the same donor have been pooled.

**Table 2:** Experimental Design.

Media	timepoint	*Donors (Human samples)*								*Animals*
		H1	H26	H27	H29	TX06	TX08	TX25	Total	M1-M5
CTR	24 h	1	1	1	1	1	1	1	**7**	**5**
	48 h	0*	1	1	1	1	1	1	**6**	**5**
G	24 h	1	1	1	1	1	1	1	**7**	**5**
	48 h	1	1	1	1	0**	1	0**	**5**	**5**
F	24 h	1	1	1	1	1	1	1	**7**	**5**
	48 h	1	1	1	1	0**	1	1	**6**	**5**
GF	24 h	1	1	1	1	1	1	1	**7**	**5**
	48 h	1	1	1	1	0**	1	1	**6**	**5**
GFI	24 h	1	1	1	1	1	1	1	**7**	**5**
	48 h	1	1	1	1	0**	1	1	**6**	**5**
GFIO	24 h	0*	1	1	1	1	1	0*	**5**	**5**
	48 h	1	1	1	1	1	1	0*	**6**	**5**
GFIP	24 h	1	1	1	1	1	1	1	**7**	**5**
	48 h	1	1	1	1	1	1	1	**7**	**5**
GFIPO	24 h	1	1	1	1	0*	0**	0**	**4**	**5**
	48 h	1	1	1	1	0*	1	1	**6**	**5**
**Total**		**14**	**16**	**16**	**16**	**10**	**15**	**12**	**99**	**80**

Sample and experimental overview. For the human study we used PCLSs from 7 human donors which have been incubated across 2 × 8 assay conditions giving a total of 112 samples. For 6 samples, the PCLSs were not suitable for RNA preparation (indicated by *). The remaining 106 samples have been processed and sequenced of which 7 samples have been identified as outliers and discarded from further analysis (indicated by **). For the mouse study we used fresh livers from five animals, which gives a total number of 16 × 5 = 80 samples. To yield enough material, three slices per sample condition have been incubated together. All mouse samples have been included. We prepared eight different media i.e. CTR: Control with 25 mM Glucose, G: 36 mM Glucose, F: 25 mM Glucose + 5 mM Fructose, GF: 36 mM Glucose + 5 mM Fructose, GFI: GF + 1 nM Insulin, GFIO: GFI + 480 μM Oleic acid, GIP: GFI + 240 μΜ Palmitic acid, GFIPO: GFI + 480 μΜ Oleic acid + 240 μΜ Palmitic acid) and cultured PCLSs from each donor for 24 h and 48 h.

As expected, freshly preserved samples from the mouse showed generally a much better RNA quality compared to the human samples. Consistently, the observed mouse RNASeq data variability is strongly driven by the experimental factors i.e. by time in culture and the different nutritional condition. In contrast, the human data shows a much stronger variability across the different liver organs which superimpose the variability implied by the experimental design. However, the balanced and paired design allows to correct for dominant confounding factors i.e. donor variability and RNA quality and to derive large gene signatures of differentially expressed genes across the different experimental conditions.

Further downstream, the integrated analysis of the two data sets allows to investigate species differences and common regulatory pathways in steatosis and liver inflammation in human and mice *ex vivo*. Exemplified we show that several strongly conserved genes are consistently up- or down-regulated with time in culture and that other genes and pathways are strongly regulated with steatosis. In addition, we also show example expression pattern of genes with opposite regulation in human vs. mouse and the expression pattern for a few well-known biomarker genes for extracellular remodeling.

In summary, the present study provides a useful and comprehensive resource for research on fatty liver diseases as it allows to interpret results from *ex vivo* experiments and to properly design new PCLSs assays for research on NAFLD and other liver diseases in the future.

## Methods

### Human study

Small human liver wedges of an equivalent size of approximately 10 g were collected from 7 human donors following partial resection, donor liver resizing or when livers were unsuitable for transplantation (see Table 1). The study was approved by the Medical Ethical Committee of the University Medical Centre Groningen (UMCG), according to Dutch legislation and the Code of Conduct for dealing responsibly with human tissue in the context of health research (www.federa.org), refraining the need of written consent for ‘further use’ of coded-anonymous human tissue. The procedures were carried out in accordance with the experimental protocols approved by the Medical Ethical Committee of the UMCG. Liver tissue was stored in University of Wisconsin preservation solution (UW, 4 °C). The total cold ischemic time was between 3 and 29 h (see Table 1). Slice viability for each donor liver was tested after one hour of culture by checking ATP production as previously described^9^. Intracellular triglyceride (TG) content of the slices was measured by a Trig/GB kit (Roche Molecular Biochemicals, Almere, the Netherlands). At the end of the experiment, PCLSs were snap-frozen and stored at −80 °C. Only non-fibrotric liver organs have been included in the present study as assessed by histopathological experts. In depth sample level histopathology data was not collected for the present study and cannot be provided here. However, the variability of steatosis within each liver organ was high and the baseline TG levels of the individual wedge biopsy are regarded as a good quantitative estimate for the degree of steatosis in the corresponding PCLSs at baseline.

### Animal study

Male C57/BL6 (Centrale Dienst Proefdieren, UMCG, Groningen, The Netherlands) were housed under standard conditions with chow and water ad libitum. Experiments were approved by the Animal Ethical Committee of the University of Groningen (Permit number 171290-01-001). At the time of sacrifice, mice were aged 10 weeks and their mean body weight was 26.4 g. Livers were harvested after exsanguination under isoflurane/O2 anesthesia and stored in UW. Liver organs from five mice have been used for PCLS preparation. A sample overview for the experiment is given in Table 2.

### PCLSs preparation and culture

Biopsies (6 mm diameter) were made from liver tissues and cut into approximately 250 μm thick slices (wet weight 5 mg) using a Krumdieck tissue slicer (Alabama Research and Development, Munford, USA) according to the protocol described^9^. All PCLSs had a wet weight of ~5 mg. PCLSs were cultured at 37degC under 80% O2 and 5% CO2, as previously described^10^. Culture lasted 24 or 48 hours and the culture medium was refreshed every 24 hours. Different culture media were used to mimic metabolic abnormalities. The control medium consisted of Williams medium E with Glutamax (Invitrogen, Bleiswijk, The Netherlands), 50 mg/mL gentamycin (Invitrogen), and 25 mM glucose (Merck, Darmstadt, Germany). Hyperglycemia was simulated by addition of 36 mM glucose or 5 mM fructose (Merck). Metabolic disorder was further mimicked by supplementation of human insulin (Sigma-Aldrich, St Louis, MO, USA), palmitic acid (Sigma-Aldrich) and oleic acid (Sigma-Aldrich) with the concentrations shown in Table 1.

### RNA isolation and quality control

Three PCLS slides per donor per assay condition have been pooled to ensure to get enough material for RNA sequencing resulting in a theoretical sample number of 112 for the human experiment and 80 samples for the mouse experiment. For the human experiment, PCLSs for 107 of 112 samples could be generated whereas for the mouse experiment, all 80 samples could be prepared. After homogenization and lysis using Qiazol buffer (Qiagen, Venlo, the Netherlands), samples were mixed with 1/5th volume of chloroform (Sigma) and separated using phase extraction tubes. The RNA was then isolated with the RNeasy Lipid Tissue Mini Kit (Qiagen, Venlo, the Netherlands) or the FavorPrep TM Tissue Total RNA Mini Kit (Favorgen, Vienna, Austria). RNA samples were quantitatively and qualitatively assessed using the fluorescence-based Broad Range Quant-iT RNA Assay Kit (ThermoFisher) and the Standard Sensitivity RNA Analysis DNF-471 Kit on a 96-channel Fragment Analyzer (Agilent), respectively. For human PCLS, concentrations averaged at 60.5 ng/µL while RIN ranged from 2.3 to 10.0, with a median at 8.0. The concentrations for mouse PCLS samples averaged at 160.3 ng/µL while RIN ranged from 6.5 to 9.0 with a median at 8.0. The RNA amount and quality was sufficient for further processing, except for one human sample which was excluded due to low RNA yield (for sample overview see also Table 2).

### Whole transcriptome profiling with ribosomal depletion

Total RNA was normalized on the MicroLab STAR automated liquid platform (Hamilton). 100 ng input was used for ribosomal RNA depletion with the QIAseq FastSelect rRNA removal Kit for Human/Mouse/Rat (Qiagen). This highly efficient rRNA removal kit resulted in a final representation of <5% rRNA, thereby negatively enriching for other RNA species such as mRNAs. We then proceeded with library construction using the NEBNext Ultra II Directional RNA Library Prep Kit for Illumina #E7760 and the NEBNext Multiplex Oligos for Illumina #E7600 (all New England Biolabs). Fragmentation time was set to 15 min. A total of 12 PCR cycles was used for the index PCR while the final libraries were eluted in 25 µL. Of note, the only deviation from the manufacturer’s protocol was the use of Ampure XP beads (Beckman Coulter) at the double-stranded cDNA purification step, instead of the recommended SPRIselect Beads. Total RNA sequencing libraries were quantified by the fluorescence dye-based methodology High Sensitivity dsDNA Quanti-iT Assay Kit (ThermoFisher) on a Synergy HTX (BioTek). Library molarity averaged at 100.3 nM. Total RNA libraries were also assessed for size distribution and adapter dimer presence by the High Sensitivity NGS Fragment DNF-474 Kit on a 96-channel Fragment Analyzer (Agilent). Sequencing libraries were normalized on the MicroLab STAR (Hamilton), pooled, and spiked in with PhiX Control v3 (Illumina). The library pool was subsequently clustered on an S2 Flow Cell and sequenced on a NovaSeq 6000 Sequencing System (Illumina) with dual index, paired-end reads at 2 × 100 bp length (Read parameters: Rd1: 101, Rd2: 8, Rd3: 8, Rd4: 101) reaching a target depth of approximately 60 million Pass-Filter reads per sample (6.5% CV, see).

### Data analysis

A detailed description of the RNASeq analysis pipeline is given in^11^. In brief, reads passing quality control filter were mapped against GRCh38 (human data) or GRCm38 (mouse data) reference genomes using STAR (v2.5.2b). Gene expression levels were quantified based on genome annotation files from Ensembl version 86 using RSEM (v1.3.0) and featureCounts (v1.5.1). For additional quality control we used FastQC (v0.11.5), picardmetrics (v0.2.4) and dupRadar (v1.0.0).

Further downstream, we observed some co-variation of the first principal components (based on all genes) with experimental confounding factors. This was partially, but not exclusively explained by low RNA RIN values.

Based on PCA we identified 7 human samples as outliers and excluded these samples from further analysis (G, F, GF, & GFI condition after 48 h from Donor TX06, GFIPO 24 h from donor TX08, and G 48 h and GFIPO 24 h from donor TX25). For the mouse experiment, all mouse samples have been included (see also Table 2 for a complete overview). For proof-of-concept we calculated differential expression in treated vs. untreated slices using limma/voom based on mapped counts with a paired donor design. As cut-off for differentially expressed genes we used |log2 fold change| > 0.5 and adjusted p-value p_adj_ ≤ 0.05. For each gene and condition, the sign of the differential expression was calculated according to sign(contrast) = 0 if p > 0.05, sign(log2 fold change) if p_adj_ ≤ 0.05.

The R-code to generate all figures is given in the Supplementary Information (Supplementary Code).

## Data Records

The raw data (FASTQ) and normalized read count and transcript per million (TPM) based processed data from the human and mouse RNASeq experiment are available in the Gene Expression Omnibus. The human and mouse experiment can be found under the accession for the super series GSE200419^12^ which refers to the individual records i.e. GSE200418 (human) and GSE200408 (mouse).

The differential expression data for all genes which have been detected in any condition (counts > 10) are available via figshare^13^.

## Technical Validation

**Fig. 1:**
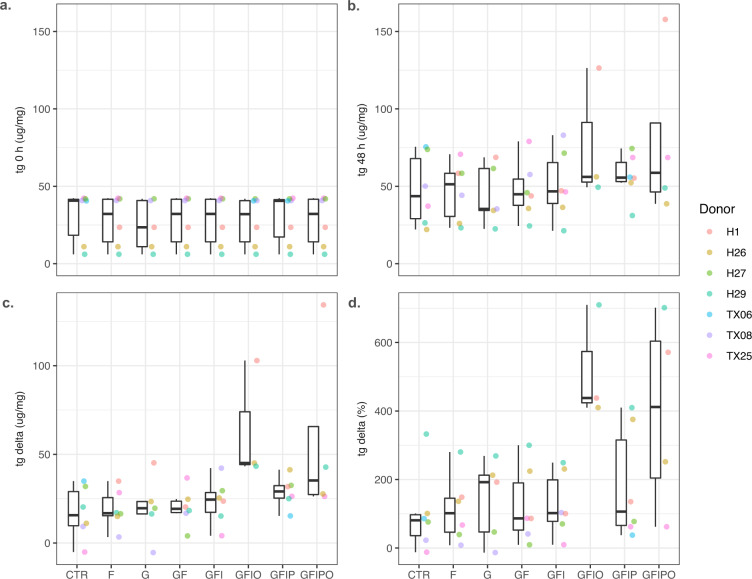
Intracellular triglyceride content of human samples. Intracellular triglyceride (tg) content (μg mg^−1^) of the human samples (**a**) before incubation (tg 0 h), (**b**) 48 h after incubation (tg 48 h), (**c**) the difference tg 48 h – tg 0 h, and (**d**). relative difference (tg 48 h – tg 0 h/tg 0 h). Colored dots indicate values per donor. Box plots indicate minimum, lower quartile, median, upper quartile, maximum value per condition.

Intracellular triglyceride (TG) content has been used as the major phenotypic readout and surrogate marker for steatosis to optimize and validate the PCLS assay. Accordingly, we measured TG content in every condition before and at the end of the 48 h culture time. As shown in Table 1 and Fig. 1, the variability of the baseline TG levels was quite high with a strong donor component. Slices from two donors have generally very low TG levels < 20 μg mg^−1^ whereas levels from four donors are 40 μg mg^−1^ or higher. Interestingly, the variability decreased quite dramatically after 48 h in culture media with a general trend of increasing levels vs. baseline conditions and strong relative increase with increasing supplementation of sugars and fatty acids.

We checked if the variability in TG content at baseline condition was correlated with RNA quality, but this was not the case. Nevertheless, the variability of the RNA quality across human samples was quite high compared to the mouse samples and there was a lower RNA yield with the human samples compared to mouse and a trend with lower yield with increasing nutrient supply (see Fig. S1). Although most human samples and all mouse samples had quite good RNA integrity numbers (RIN) >7.5, several human samples had sub-optimal RNA quality for standard RNA preparation using polyA enrichment of full-length transcripts. Therefore, we applied total RNA preparation protocol for both studies. Interestingly, the RIN numbers showed a slight increase with time in culture, however there was no further systematic bias introduced by the different culture conditions.

**Fig. 2:**
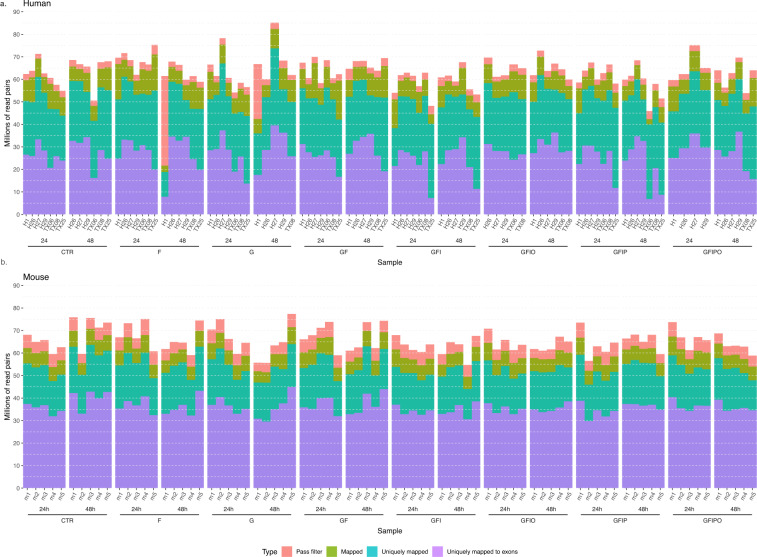
Sequencing metrics from RNASeq data analysis. Number of reads per human (**a**) and mouse (**b**) sample according to quality metrics from the analysis pipeline. Each column bar shows the cumulative number of reads that map (1) uniquely to coding regions (uniquely mapped to exon), (2) uniquely to any region (uniquely mapped), (3) map to genome including ambiguous mappings (map), and (4) that passed the quality control filter (Pass filter).

We sequenced the human and mouse samples with a relatively high coverage of >60 million paired-end reads per sample on average because we expected a lower rate of uniquely mapping reads for protein coding regions compared to a standard mRNA protocol. As shown in Fig. 2, more than 50% of these reads did not map uniquely to the corresponding genomes whereby the variability of uniquely mapped reads per sample was higher in the human study and the fraction of uniquely mapped genes was smaller compared to the mouse study. However, we obtained a quite deep sequencing profile from all the samples. A large proportion of the uniquely mapped reads belong to non-protein-coding genes as shown in detail in Fig. S2. Of note, the different fractions of non-protein coding genes in the human study vs the mouse study can be at least partially explained by the deeper annotation of the human genome with 38,060 non-coding genes compared to 26,691 non-coding genes in the mouse genome, according to our reference genome annotation Ensembl 86 (see Methods). In principle, the present data set allows to explore expression patterns across all these biotypes. Again, the mouse data set shows a homogenous distribution of the number of detected genes per biotype and assay condition compared to the human data set where there is a drop in number of genes at G 48 h, GFIO 48 h and GFIPO 24 h condition.

**Fig. 3:**
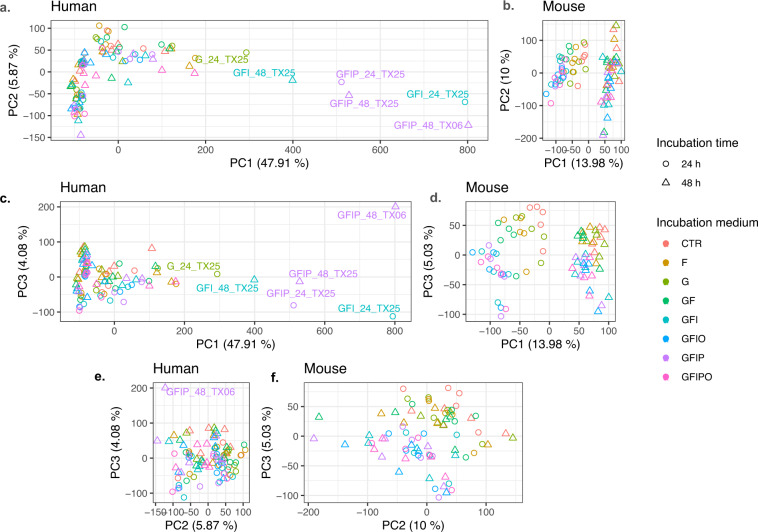
Principal Component Analysis. Scatter plot with of 1^st^ principal component (PC) vs. 2^nd^ PC (**a,b**), 1^st^ vs. 3^rd^ (**c,d**) and 2^nd^ vs. 3^rd^ (**e,f**) for human (**a,c,e**) and mouse (**b,d,f**) samples. Different symbol shapes indicate different timepoints i.e. circles for 24 h and triangles for 48 h. Different colors indicate the different conditions. All plots have the same height and 1:1 scale of both axes, aspect ratio of individual plot is determined by relative spread across the two principal components.

We investigated the major factors driving the variability in the two data sets using Principal Component Analysis (PCA). Scatter plots for all samples for the first three dimensions colored by condition and time in culture are shown in Fig. 3 (see also Fig. S3) with alternative coloring by donor and time). In addition, we explored the coefficient of variant across the different conditions (Fig. S4). PC1 of the human samples is widely spread by six individual samples with a high PC1 value whereas PC2 is mostly driven by timepoint. PC3 is rather scattered but shows some clustering with regard to culture medium. PC1 explains almost 50% of the observed variance whereas the remaining PCs contribute with less than 10% to the overall explained variance. In contrast, the mouse samples cluster nicely with regard to the two major experimental design factors i.e. with time (PC1) and culture media (PC2 and PC3). The fraction of explained variance of the first two PCs is less than 30% in total (see Fig. S5).

## Biological Validation

**Fig. 4:**
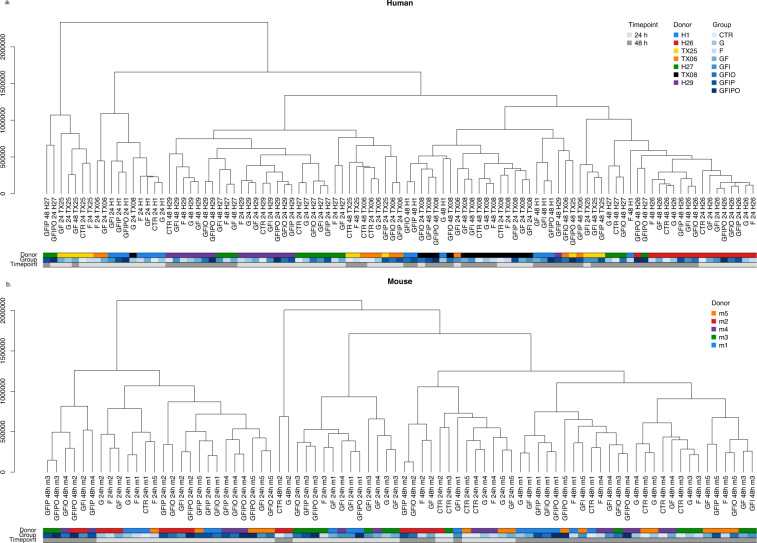
Hierarchical clustering. Hierarchical clustering of human (**a**) and mouse (**b**) samples. The dendrograms show the samples grouped according to Euclidean distance and complete linkage using the voom- and log-transformed gene counts per million.

We extracted the genes with the highest PC loadings in the human and mouse data sets, respectively (see Fig. S6). Altogether, the human and mouse data look quite different based on the readouts from the PCA. This is further confirmed by hierarchical clustering of the individual samples (Fig. 4). Quite consistent with the PCA, the mouse samples nicely correlate with regard to the major experimental factors (time and culture media). In contrast, the human samples show a strong donor bias and most samples cluster first by donor organ. Accordingly, donor batch correction of the human data is essential to explore common or species-specific differential expression patterns with the different assay conditions.

**Fig. 5:**
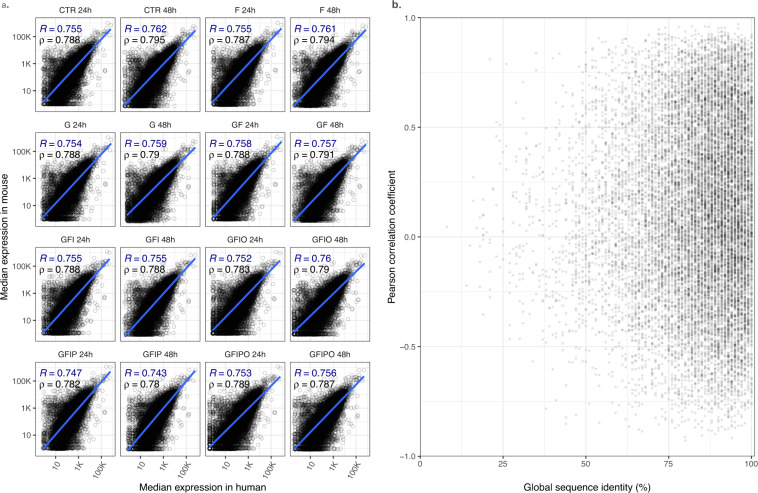
Human vs. mouse gene expression correlation. Human vs. mouse gene expression correlation. (**a**) Correlation of mouse vs. orthologous human gene expression (log-scale) per sample condition. All mouse genes with their defined next human ortholog have been included. The blue line and coefficient show log-linear correlation line based on Pearson’s R while the black coefficient shows the correlation coefficient based on Spearman’s coefficient. (**b**) Scatter plot of expression correlation coefficient vs. the global sequence identity for all pairs of homologous genes.

Having this in mind, the correlation of the batch corrected gene expression data of human vs. corresponding orthologous mouse genes shows quite reasonable agreement. We further explored this by testing whether highly conserved genes show higher expression correlation (Fig. 5), which is only partially the case in our data. However, we also used this approach to select genes with a very high expression correlation across the different assay conditions. As shown in Table 3, the top 15 correlated genes show a very high expression correlation with R > 0.92 and most of them are also highly conserved.

**Table 3:** Top15 genes based on expression correlation.

Gene symbol	Pearson R	% global identity	Human ensgid	Mouse ensgid
FBN1	0.97	96	ENSG00000166147	ENSMUSG00000027204
MT1A	0.96	82	ENSG00000205362	ENSMUSG00000031765
CNNM3	0.96	86	ENSG00000168763	ENSMUSG00000001138
MAGED2	0.96	87	ENSG00000102316	ENSMUSG00000025268
STMN1	0.96	78	ENSG00000117632	ENSMUSG00000028832
COL6A3	0.95	74	ENSG00000163359	ENSMUSG00000048126
DDX56	0.95	91	ENSG00000136271	ENSMUSG00000004393
BICC1	0.95	92	ENSG00000122870	ENSMUSG00000014329
COL1A2	0.95	90	ENSG00000164692	ENSMUSG00000029661
MMP2	0.95	96	ENSG00000087245	ENSMUSG00000031740
ACKR2	0.95	72	ENSG00000144648	ENSMUSG00000044534
PTRF	0.94	83	ENSG00000177469	ENSMUSG00000004044
SMPDL3A	0.94	80	ENSG00000172594	ENSMUSG00000019872
COL3A1	0.94	91	ENSG00000168542	ENSMUSG00000026043
ERBB3	0.93	91	ENSG00000065361	ENSMUSG00000018166

Top15 genes with highest correlation of group median expression in human vs. mouse tissues.

**Fig. 6:**
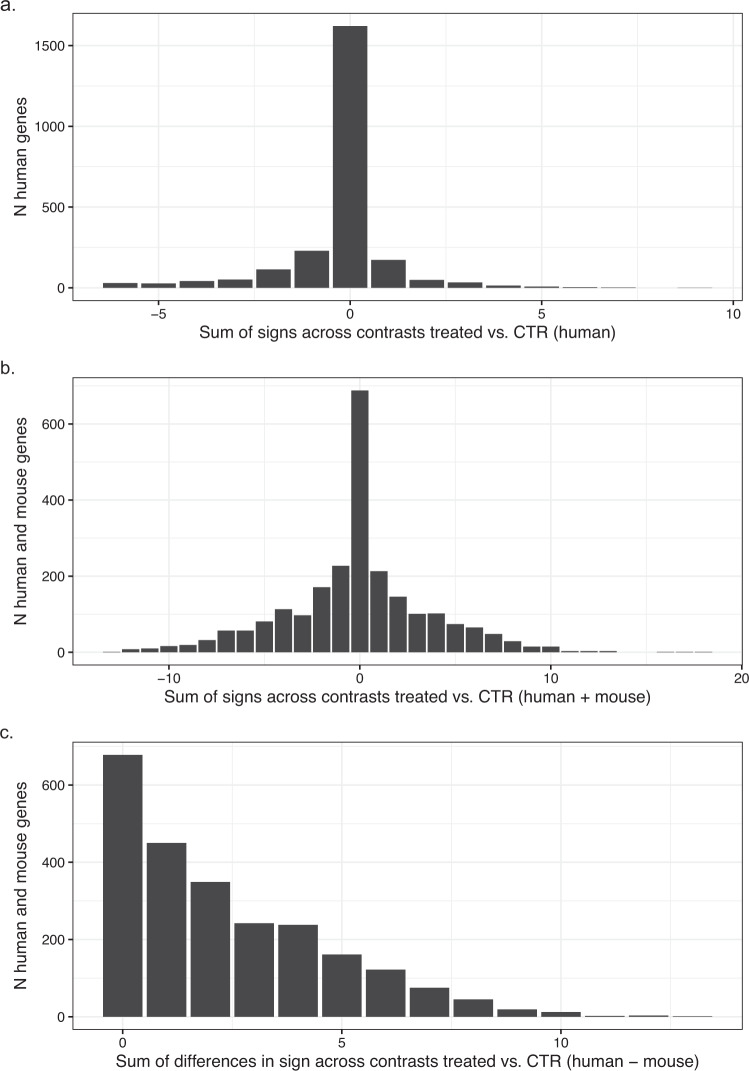
Top regulated genes based on experimental design. Top regulated genes based on experimental design. For each nutrient supplementation condition, the differential expression contrast was calculated using the expression with nutrient supplementation as test condition vs. time matched baseline condition as the control condition (e.g. Glucose vs. CTR at 24 h). The three histograms show the integrated distribution across all contrasts i.e. number of genes by (**a**) sum of signs across the all 14 human contrasts; (**b**) sum of signs in the combined 28 human + mouse contrasts; and **c**. the sum of absolute differences in signs of the human vs. the corresponding mouse contrast. For a given contrast, a gene is considered as being differentially expressed vs. control condition if |log-ratio| ≥0.5 and padj ≤0.05. The integrated sum of sign is calculated by counting up-regulated conditions as 1 and down-regulated condition as −1. Maximum and minimum sums correspond to +14 and −14 per species (**a**), +28 and −28 for the combined experiment (**b**) and 0, 28 for the sum of different signs, respectively.

In a second approach, we wanted to identify genes which show a similar differential expression pattern across the different assay conditions. For this exercise, we scored the conditions which showed up- or down- regulation for each gene with +1 or −1, respectively, as condition score and ranked all genes with regard to the integrated sum of condition scores across all human and mouse conditions. The histogram in Fig. 6a shows the total score distribution across all genes in the human experiment. From this histogram we selected all genes with absolute total score equal or greater than 8 and ranked those genes by the integrated condition score across the combined human and the mouse conditions (Fig. 6b). The integrated total condition score takes into account the magnitude and significance level of the differential expression and thus prioritizes genes which show a strong transcriptional response with regard to the experimental design i.e. the different culture media. The top 18 genes based on this ranking are shown in Table 4. As a third approach, we wanted to search for genes with a poor agreement in the human vs. mouse experiment. Accordingly, we calculated for each gene the number of conditions with opposite differential expression sign in the human vs. corresponding mouse condition (Fig. 6c) and selected the genes with the highest divergent condition scores as examples of genes with poor species conservation and species differences.

**Table 4:** Top18 genes based on experimental design.

gene_symbol	sum_score_h	ensgid	mouse_ensgid
FST	94.7538168	ENSG00000134363	ENSMUSG00000021765
ARRDC4	67.8910773	ENSG00000140450	ENSMUSG00000042659
DEPP1	63.3306733	ENSG00000165507	ENSMUSG00000048489
PNPLA5	55.1928157	ENSG00000100341	ENSMUSG00000018868
FADS2	39.3079891	ENSG00000134824	ENSMUSG00000024665
FASN	34.9093403	ENSG00000169710	ENSMUSG00000025153
ERBB3	32.1739147	ENSG00000065361	ENSMUSG00000018166
YPEL3	30.926969	ENSG00000090238	ENSMUSG00000042675
PCK1	29.4629409	ENSG00000124253	ENSMUSG00000027513
MID1IP1	27.9817927	ENSG00000165175	ENSMUSG00000008035
LPIN2	27.1569665	ENSG00000101577	ENSMUSG00000024052
ATG2A	24.6063739	ENSG00000110046	ENSMUSG00000024773
ABCA9	24.3030494	ENSG00000154258	ENSMUSG00000041797
OCLN	23.7841083	ENSG00000197822	ENSMUSG00000021638
GRB7	23.4696009	ENSG00000141738	ENSMUSG00000019312
ANGPTL8	23.4366564	ENSG00000130173	ENSMUSG00000047822
HSD17B11	22.3194899	ENSG00000198189	ENSMUSG00000029311
NR1I3	20.2573204	ENSG00000143257	ENSMUSG00000005677

Top 18 genes based on consistent differential expression across different conditions (abs(“sum of sign”) in treated vs. CTR for human and mouse conditions >8) ranked by differential expression in human data set (sum_score_h = abs(sum(LogRatio * -log10padj across conditions).

**Fig. 7:**
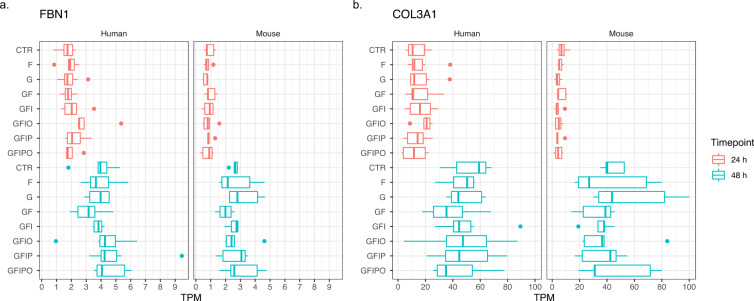
Expression pattern of top correlated genes. Expression of two representative genes with high expression correlation and sequence similarity in human and mouse. (**a**) FBN1 (fibrillin-1, ENSG00000166147) and (**b**) COL3A1 (Collagen alpha-1(III) chain, ENSG00000168542) have been selected from Table 3 based on high expression correlation across PCLSs and high protein sequence similarity between human and mouse. Box plot indicates minimum, lower quartile, median, upper quartile, maximum value per condition.

**Fig. 8:**
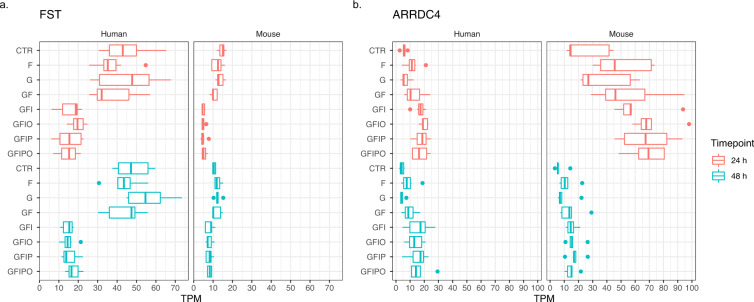
Expression pattern of top regulated genes. Expression of two representative genes with strong and consistent transcriptional regulation across assay conditions. (**a**) FST (follistatin, ENSG00000134363) and (**b**) ARRDC4 (arrestin domain containing 4, ENSG00000140450) have been selected from Table 4 as representative examples of genes which show a strong and consistent transcriptional regulation across the assay conditions with a strong expression decrease or increase with incremental nutrient supplementation for FST and ARRDC4, respectively. Box plot indicates minimum, lower quartile, median, upper quartile, maximum value per condition.

Interestingly, the two different methods for identifying conserved genes with a similar expression pattern in both experiments (i.e. Table 3 vs. Table 4) led to the enrichment of quite different predominant gene expression patterns (Figs. 7, 8). The top genes based on expression correlation show a clear time dependent expression pattern with a strong in- or decrease of expression with time in culture like representative genes FBN1 and COL3A1 gene for this type of expression pattern (see Fig. 7). Also it is worthy to note that Table 3 lists a number of additional collagen protein coding genes i.e. COL1A2 and COL6A3 with this type of expression pattern pointing to general remodeling processes of the extracellular matrix that is taking place in the PCLS assay and that has been extensively described previously^8^. In contrast to the top genes based on expression correlation (Table 3), the top genes across the assay conditions (Table 4) are stronger affected by different culture media and show an increase or decrease with incremental supplementation of sugar and fatty acids as e.g. shown for FST and ARRDC4 (see Fig. 8). However, the expression pattern for murine ARRDC4 shows that both types of patterns can occur i.e. a change with time in culture (decrease from 24 h to 48 h) and an increase with sugar and fatty acid supplementation pointing to strong connections between the underlying molecular processes.

**Fig. 9:**
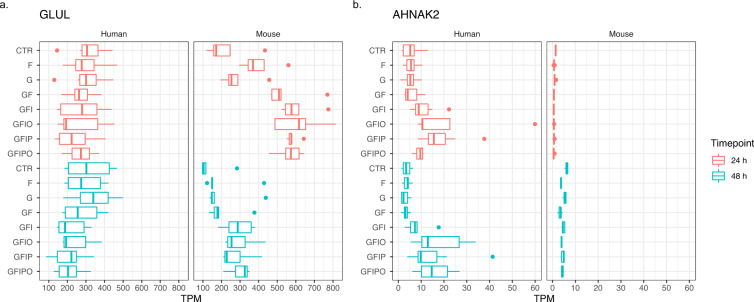
Expression pattern of top genes with species differences. Expression of two representative genes that show opposite patterns in human and mouse. (**a**) GLUL (glutamate-ammonia ligase, ENSG00000135821) and (**b**) AHNAK2 (AHNAK nucleoprotein 2, ENSG00000185567) have been selected as representative examples of genes which show an opposite expression pattern across conditions in human and mouse. Box plot indicates minimum, lower quartile, median, upper quartile, maximum value per condition.

The present study is also quite useful to investigate species differences in the PCLS assay. Figure 9 shows two examples of genes with quite opposite differential expression pattern with time in culture and across nutrient conditions (top 2 genes ranked from Fig. 6c). GLUL encodes for the enzyme for Glutamine synthetase and has in the liver the important function to remove ammonia^14^. Whereas the human gene shows rather a decrease with nutrient supplementation and no change from 24 h to 48 h, the mouse genes shows a strong increase with nutrient supplementation, specifically with Fructose, and a decrease with from 24 h to 48 h. The second example AHNAK2 encodes for an intracellular protein with poorly described function. Here we see observe a slight increase from 24 h to 48 h for the mouse gene but no change with nutrient supplementation whereas the human ortholog gene shows a very strong increase with nutrient supplementation, specifically with insulin and fatty acid supplementation.

**Fig. 10:**
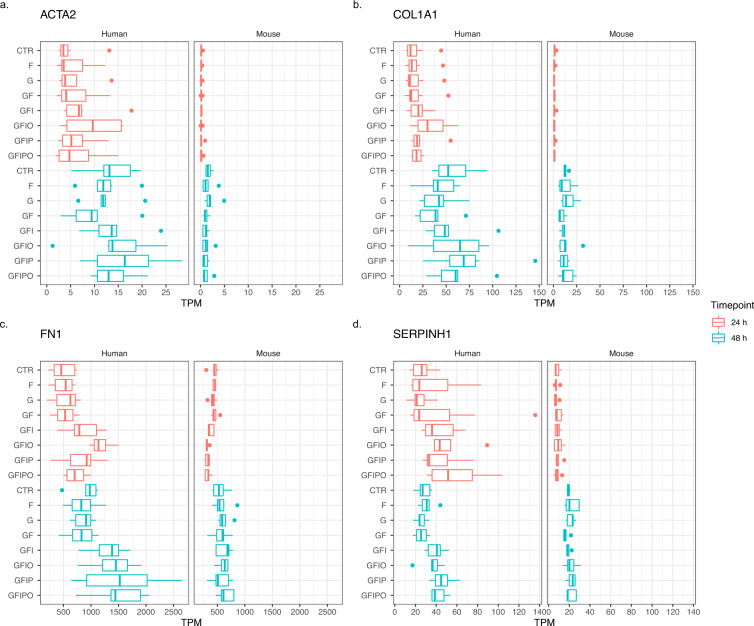
Expression pattern of selected ECM biomarker genes. Expression of four well-described biomarker genes for extracellular matrix (ECM) remodelling: (**a**) ACTA2 (Actin alpha 2, smooth muscle), (**b**) COL1A1 (collagen type I alpha 1 chain), (**c**) FN1 (fibronectin 1) and (**d**) SERPINH1 (serpin family H member 1, HSP47).

Finally, we also selected a few well-established biomarker genes for extracellular matrix (ECM) remodeling^15–17^ to further characterize our assay (Fig. 10). Consistent with previous findings, we see an increased expression with time in culture for all selected ECM markers in the human and the mouse experiment. Furthermore, the steatotic condition induced by nutrient supplementation leads to further increase of ECM marker expression highlighting the relevance for this assay for NAFLD research.

## Usage Notes

The present study provides a comprehensive description of a new *ex vivo* assay for NAFLD which allows to test anti-steatotic and anti-inflammatory drugs preclinically in human tissue samples. The human centric setup of the study is a unique feature of the assay since it allows mechanistic investigations of the disease pathology *in situ*. However, the access to fresh human tissues is strongly restricted due to ethical reasons and the sample variability is high. Accordingly, we complemented the experimental design by liver slices from young healthy mice which have been cultivated at the exact same conditions as the human slices. We think that this additional arm of the study will foster the interpretation of preclinical data on NAFLD and thus will increase their translatability.

A number of important pathways in the liver are known to be differentially regulated in humans vs. mice. For example, in contrast to humans, rodents are very prone to tumorigenesis induced by Peroxisome proliferator-activated receptors (PPAR)^18^. Also important aspects of CYP-mediated drug and bile acid metabolism are different in human and mice^19,20^. The present study allows to systematically investigate these differences under pro-inflammatory and lipogenic culture conditions and thus provides a new tool to test new NAFLD drugs. It also highlights that the PCLS assay requires only a few animals and human samples to design a complex comparative and disease relevant assay. Interestingly, the high variability of triglyceride content and other parameters observed with the human samples prior PCLS culture, partially dissolves over time. The strong time dependent and highly conserved transcriptional changes which are observed between 48 h and 24 h in all culture conditions have been characterized in detail previously^8^. The corresponding genes include a number protein coding genes for the extracellular matrix as e.g. COL1A2, COL3A1, COL6A3, FBN1, MMP2 (Table 3). The incremental supplementation of sugars and fatty acids to the PCLS culture medium triggers additional transcriptional programs which go in line with the observed increase in lipogenesis and TG synthesis. A full description and functional characterization of these conditions including quantifications of pro-inflammatory cytokines is presented in a companion paper.

## Supplementary information

Supplementary Information

## Data Availability

The custom data analysis of the present paper as well as the paper figures have been performed using RStudio Markdown and Bioconductor. A pdf version of the report including the R-Code and figures is available as Supplementary Information.

## References

[1] Zelber-Sagi S (2007). Long term nutritional intake and the risk for non-alcoholic fatty liver disease (NAFLD): A population based study. J Hepatol.

[2] Wanless IR, Lentz JS (1990). Fatty liver hepatitis (steatohepatitis) and obesity: An autopsy study with analysis of risk factors. Hepatology.

[3] Angulo P (2002). Nonalcoholic Fatty Liver Disease. New Engl J Medicine.

[4] Younossi ZM (2016). The economic and clinical burden of nonalcoholic fatty liver disease in the United States and Europe. Hepatology.

[5] Cole BK, Feaver RE, Wamhoff BR, Dash A (2017). Non-alcoholic fatty liver disease (NAFLD) models in drug discovery. Expert Opin Drug Dis.

[6] de Graaf IAM (2010). Preparation and incubation of precision-cut liver and intestinal slices for application in drug metabolism and toxicity studies. Nat Protoc.

[7] Olinga P, Schuppan D (2013). Precision-cut liver slices: a tool to model the liver *ex vivo*. J Hepatol.

[8] Bigaeva, E. *et al*. Transcriptomic characterization of culture-associated changes in murine and human precision-cut tissue slices. *Arch Toxicol* 1–35, 10.1007/s00204-019-02611-6 (2019). 10.1007/s00204-019-02611-6 31754732

[9] de Graaf IAM (2010). Preparation and incubation of precision-cut liver and intestinal slices for application in drug metabolism and toxicity studies. Nat Protoc.

[10] Prins GH (2019). A Pathophysiological Model of Non-Alcoholic Fatty Liver Disease Using Precision-Cut Liver Slices. Nutrients.

[11] Schlager S (2020). Inducible knock-out of BCL6 in lymphoma cells results in tumor stasis. Oncotarget.

[12] Simon E, Motyka M, Prins GH, Olinga P, Oldenburger A (2023). GEO.

[13] Simon E (2023). figshare.

[14] Hakvoort TBM (2017). Hepatology - 2016 - Hakvoort - Pivotal role of glutamine synthetase in ammonia detoxification.pdf. Hepatology.

[15] Busch S (2017). Cellular organization and molecular differentiation model of breast cancer-associated fibroblasts. Mol Cancer.

[16] Stribos EGD, Seelen MA, Goor H, van, Olinga P, Mutsaers HAM (2017). Murine Precision-Cut Kidney Slices as an *ex vivo* Model to Evaluate the Role of Transforming Growth Factor-β1 Signaling in the Onset of Renal Fibrosis. Front Physiol.

[17] Alsafadi HN (2017). An *ex vivo* model to induce early fibrosis-like changes in human precision-cut lung slices. Am J Physiol-lung C.

[18] Pawlak M, Lefebvre P, Staels B (2015). Molecular mechanism of PPARα action and its impact on lipid metabolism, inflammation and fibrosis in non-alcoholic fatty liver disease. J Hepatol.

[19] Martignoni M, Groothuis GMM, de Kanter R (2006). Species differences between mouse, rat, dog, monkey and human CYP-mediated drug metabolism, inhibition and induction. Expert Opin Drug Met.

[20] Takahashi S (2016). Cyp2c70 is responsible for the species difference in bile acid metabolism between mice and humans[S]. J Lipid Res.

