# Supplementary material for: Transcriptomic profiling of induced steatosis in human and mouse precision-cut liver slices
Source: Sci Data. 2023 May 19;10:304. doi: 10.1038/s41597-023-02220-0 (PMC10199090; doi:10.1038/s41597-023-02220-0)
Supplement: Supplementary file 1 — Supplementary Information [file 41597_2023_2220_MOESM1_ESM.pdf]

Supplementary Information

Figure S1: RNA quality and yield.
Box plots show RNA integrity numbers (RIN) and RNA concentration yield from all human (left) and mouse (right) samples across the different conditions of our experiment. Color code indicates the sample donor or animal. Box plot indicates minimum, lower quartile, median, upper quartile, maximum value per condition. The dashed line indicates RIN = 7.5.

Figure S2: Gene Biotype
Gene biotype distribution of mapped genes across experimental conditions as predicted by our processing pipeline and Ensembl 86 reference annotation.

Figure S3. Principal Component Analysis.
Scatter plot with of 1st principal component (PC) vs. 2nd PC (top), 1st vs. 3rd (middle) and 2nd vs. 3rd (bottom) for human (left) and mouse (right) samples. Different symbol shapes indicate different timepoints i.e. circles for 24h and triangles for 48h. Different colors indicate the different donors. All plots have the same height and 1:1 scale of both axes, aspect ratio of individual plot is determined by relative spread across the two principal components.

Figure S4. Expression variability across conditions.
Comparison of squared variation coefficient across conditions between human (left) and mouse (right) samples. Condition is marked by color code. Smoothing function is applied according to gam (generalized additive models with integrated smoothness estimation).

Figure S5. Explained variance by Principal Component.
Line plots showing the explained variance (%) by first ten principal components for human (left) and mouse (right).

Figure S6. Top Genes from PCA loadings.
Top genes based on PCA loadings in human (left) and mouse (right).

Supplementary Code.
R-Code for generating the figures and table in the manuscript.

Fig S1

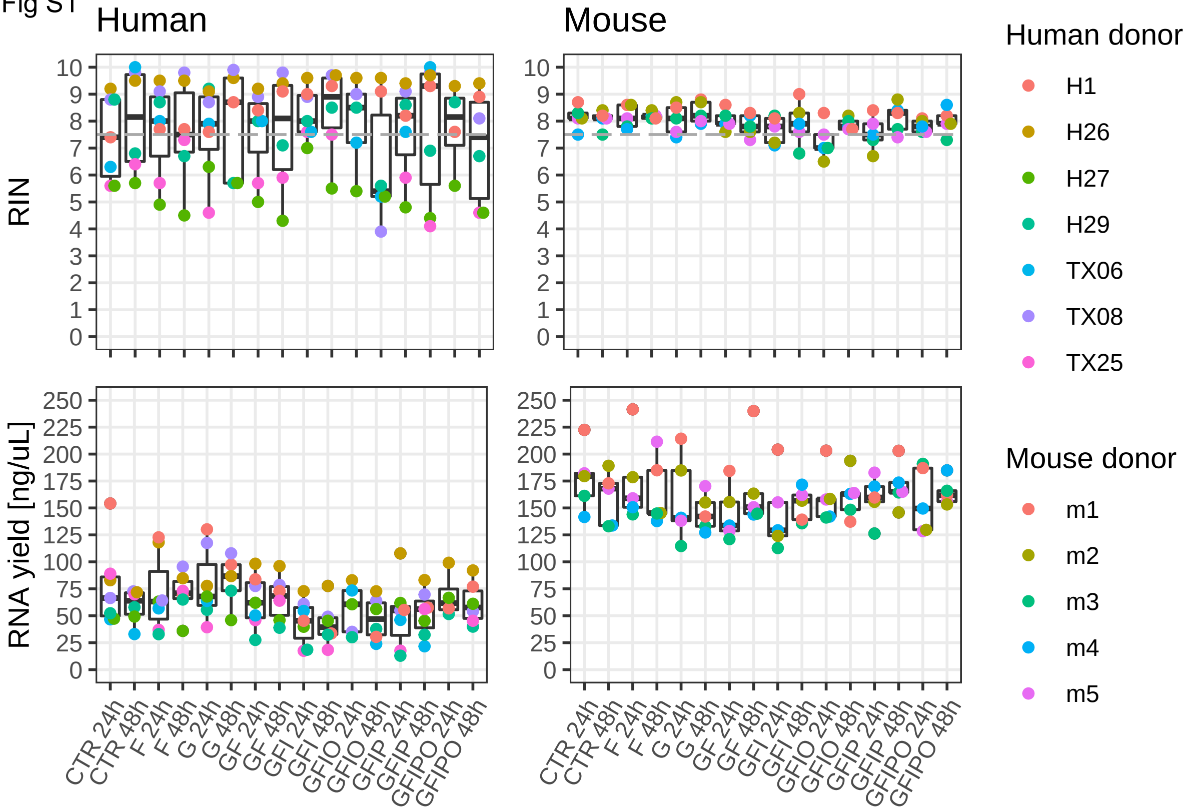

Human
Mouse
RIN
RNA yield [ng/uL]
0
1
2
3
4
5
6
7
8
9
10
0
25
50
75
100
125
150
175
200
225
250
CTR 24h
CTR 48h
F 24h
F 48h
G 24h
G 48h
GF 24h
GF 48h
GFI 24h
GFI 48h
GFIO 24h
GFIO 48h
GFIP 24h
GFIP 48h
GFIPO 24h
GFIPO 48h
Human donor
H1
H26
H27
H29
TX06
TX08
TX25
Mouse donor
m1
m2
m3
m4
m5

Fig S2

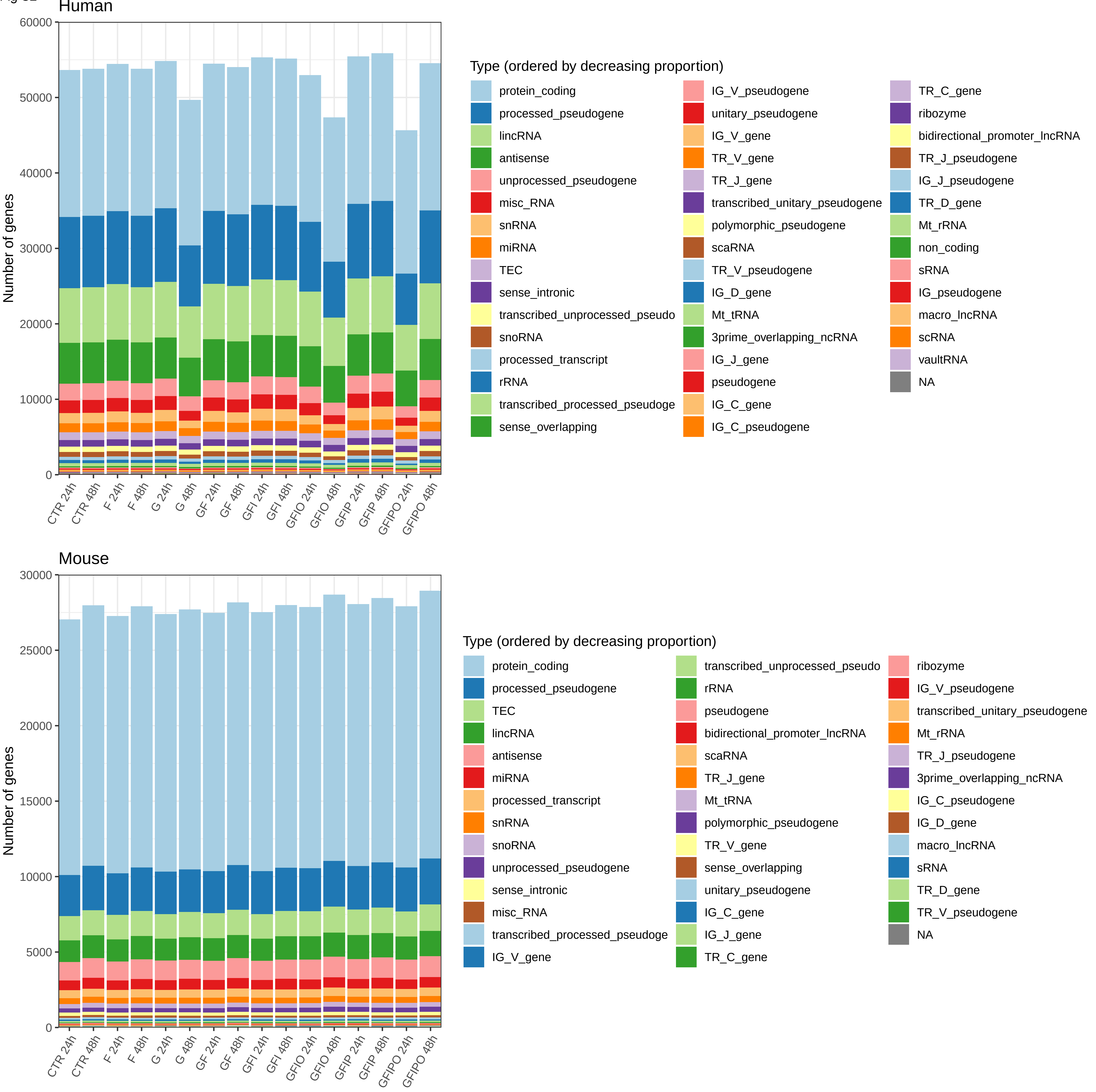
Human
Number of genes
60000
50000
40000
30000
20000
10000
0
CTR 24h
CTR 48h
F 24h
F 48h
G 24h
G 48h
GF 24h
GF 48h
GFI 24h
GFI 48h
GFIO 24h
GFIO 48h
GFIP 24h
GFIP 48h
GFIPO 24h
GFIPO 48h
Type (ordered by decreasing proportion)
protein_coding
processed_pseudogene
lincRNA
antisense
unprocessed_pseudogene
misc_RNA
snRNA
miRNA
TEC
sense_intronic
transcribed_unprocessed_pseudo
snoRNA
processed_transcript
rRNA
transcribed_processed_pseudoge
sense_overlapping
IG_V_pseudogene
unitary_pseudogene
IG_V_gene
TR_V_gene
TR_J_gene
transcribed_unitary_pseudogene
polymorphic_pseudogene
scaRNA
TR_V_pseudogene
IG_D_gene
Mt_tRNA
3prime_overlapping_ncRNA
IG_J_gene
pseudogene
IG_C_gene
IG_C_pseudogene
TR_C_gene
ribozyme
bidirectional_promoter_lncRNA
TR_J_pseudogene
IG_J_pseudogene
TR_D_gene
Mt_rRNA
non_coding
sRNA
IG_pseudogene
macro_lncRNA
scRNA
vaultRNA
NA
Mouse
Number of genes
30000
25000
20000
15000
10000
5000
0
CTR 24h
CTR 48h
F 24h
F 48h
G 24h
G 48h
GF 24h
GF 48h
GFI 24h
GFI 48h
GFIO 24h
GFIO 48h
GFIP 24h
GFIP 48h
GFIPO 24h
GFIPO 48h
Type (ordered by decreasing proportion)
protein_coding
processed_pseudogene
TEC
lincRNA
antisense
miRNA
processed_transcript
snRNA
snoRNA
unprocessed_pseudogene
sense_intronic
misc_RNA
transcribed_processed_pseudoge
IG_V_gene
transcribed_unprocessed_pseudo
rRNA
pseudogene
bidirectional_promoter_lncRNA
scaRNA
TR_J_gene
Mt_tRNA
polymorphic_pseudogene
TR_V_gene
sense_overlapping
unitary_pseudogene
IG_C_gene
IG_J_gene
TR_C_gene
ribozyme
IG_V_pseudogene
transcribed_unitary_pseudogene
Mt_rRNA
TR_J_pseudogene
3prime_overlapping_ncRNA
IG_C_pseudogene
IG_D_gene
macro_lncRNA
sRNA
TR_D_gene
TR_V_pseudogene
NA

Fig S3

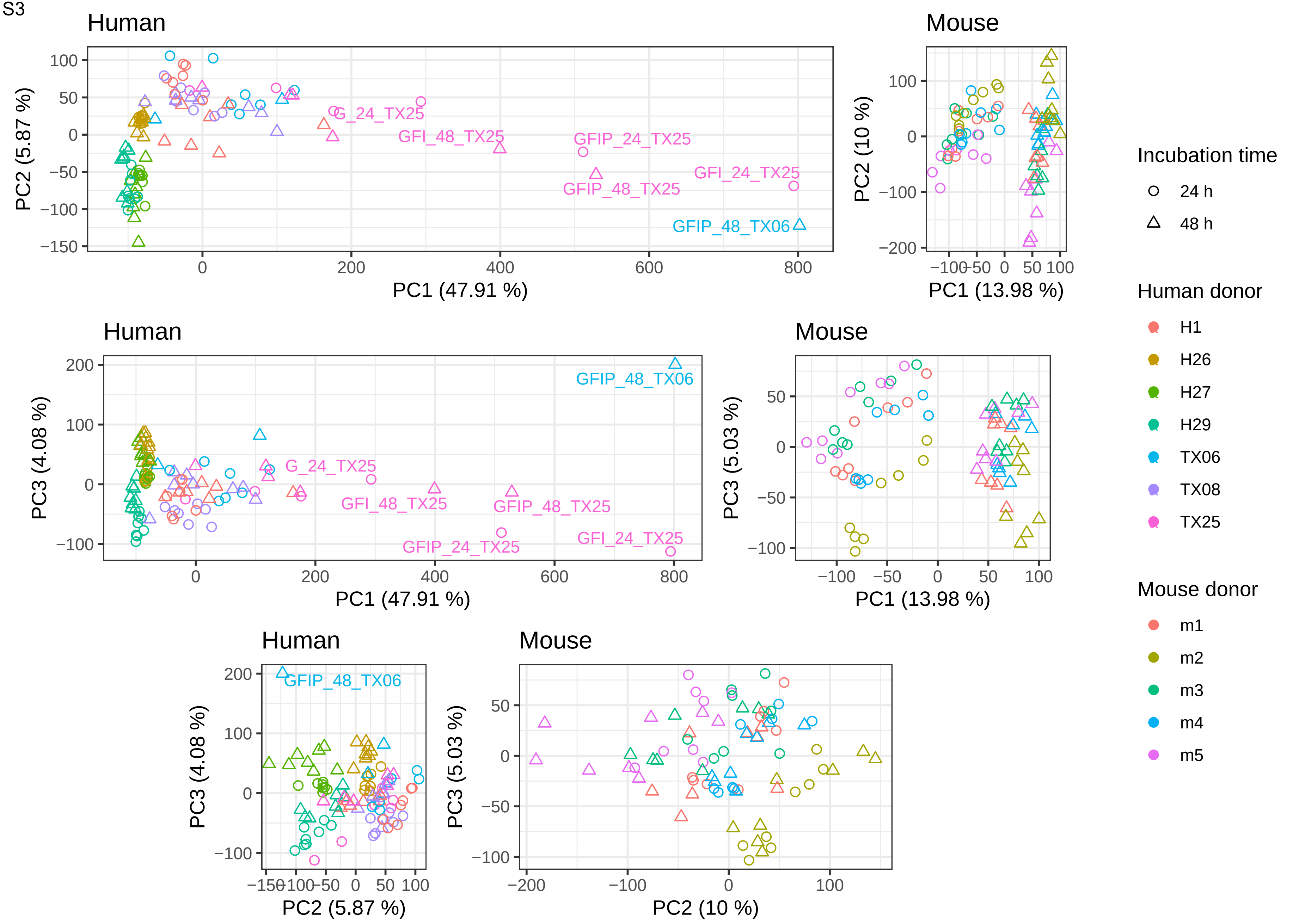
Human
Mouse
PC1 (47.91 %)
PC2 (5.87 %)
PC3 (4.08 %)
PC1 (13.98 %)
PC2 (10 %)
PC3 (5.03 %)
G_24_TX25
GFI_48_TX25
GFIP_24_TX25
GFIP_48_TX25
GFI_24_TX25
GFIP_48_TX06
Incubation time
24 h
48 h
Human donor
H1
H26
H27
H29
TX06
TX08
TX25
Mouse donor
m1
m2
m3
m4
m5

Fig S4

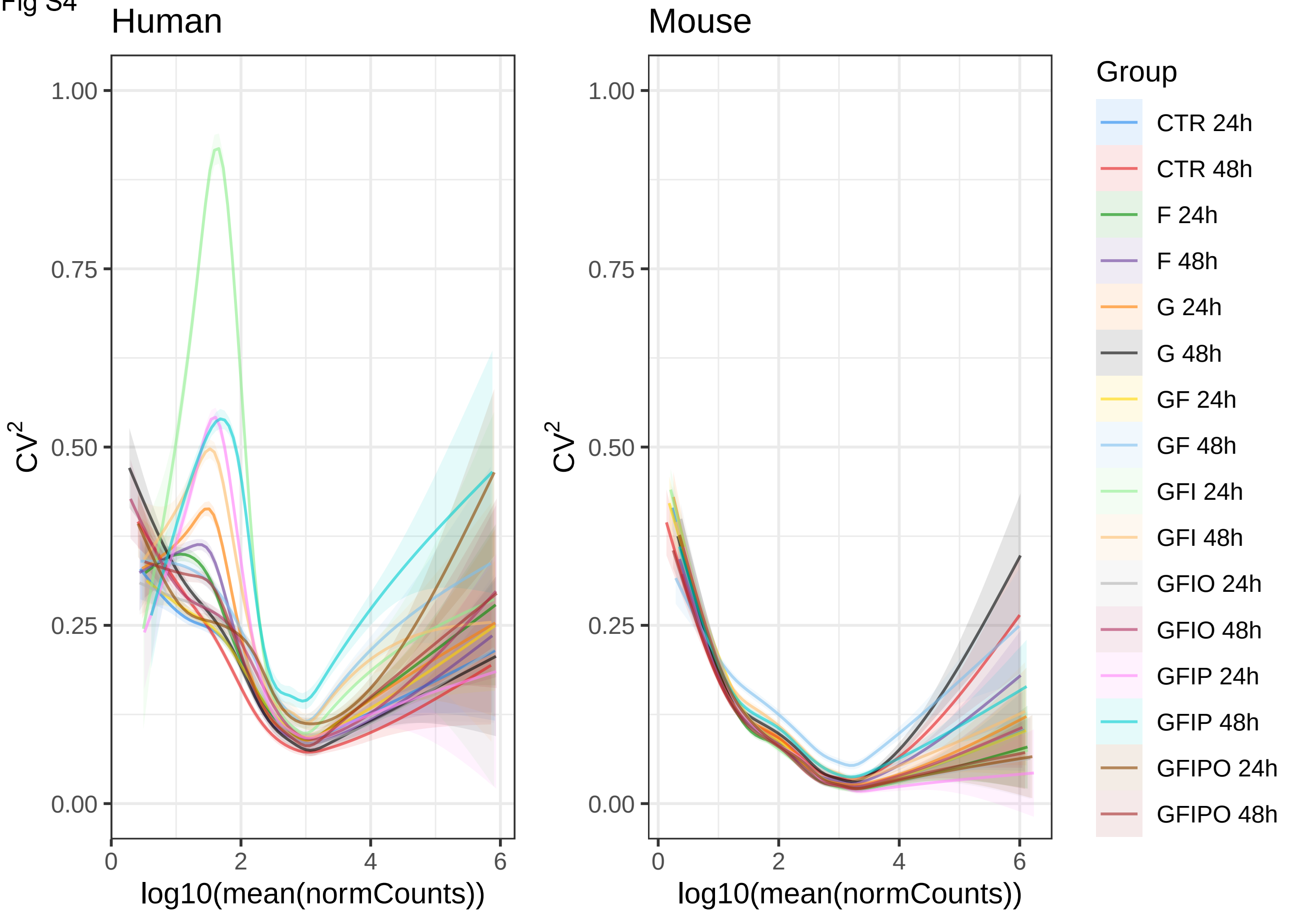

Human
Mouse
1.00
0.75
0.50
0.25
0.00
CV²
0
2
4
6
log10(mean(normCounts))
Group
CTR 24h
CTR 48h
F 24h
F 48h
G 24h
G 48h
GF 24h
GF 48h
GFI 24h
GFI 48h
GFIO 24h
GFIO 48h
GFIP 24h
GFIP 48h
GFIPO 24h
GFIPO 48h

Fig S5

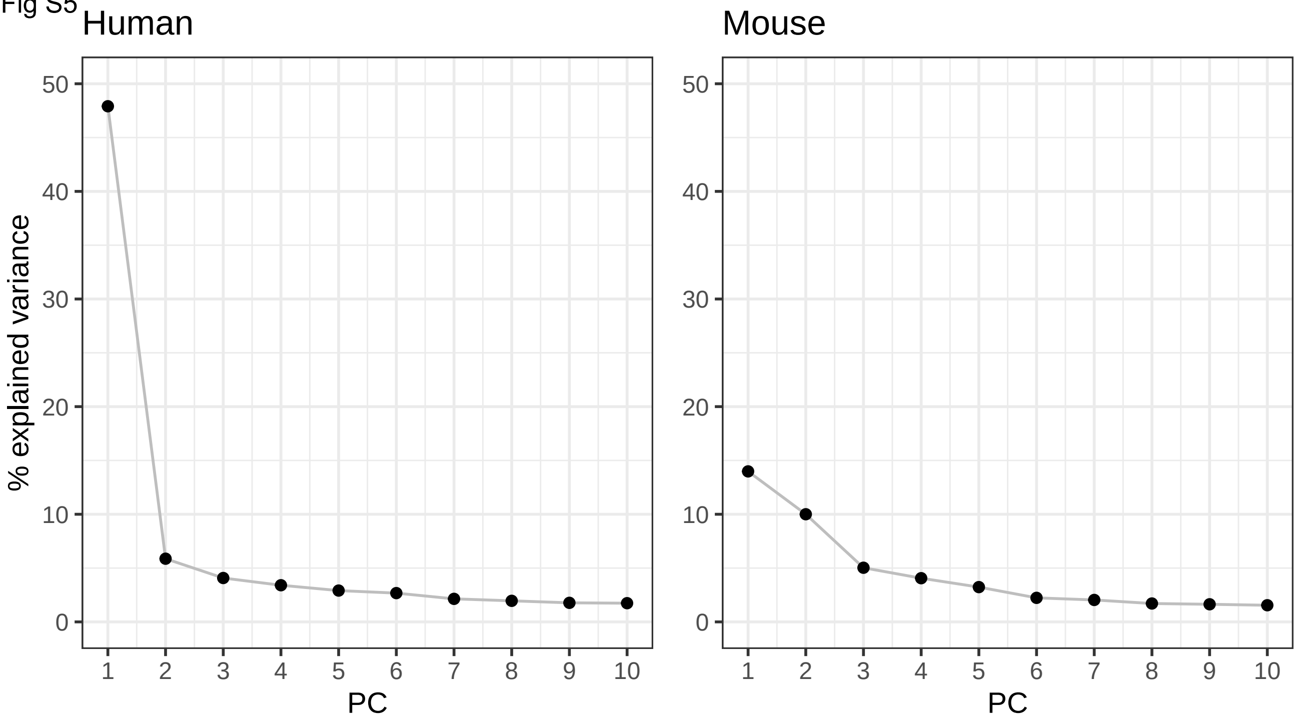
Human
Mouse
% explained variance
0
10
20
30
40
50
1
2
3
4
5
6
7
8
9
10
PC
PC

Fig S6

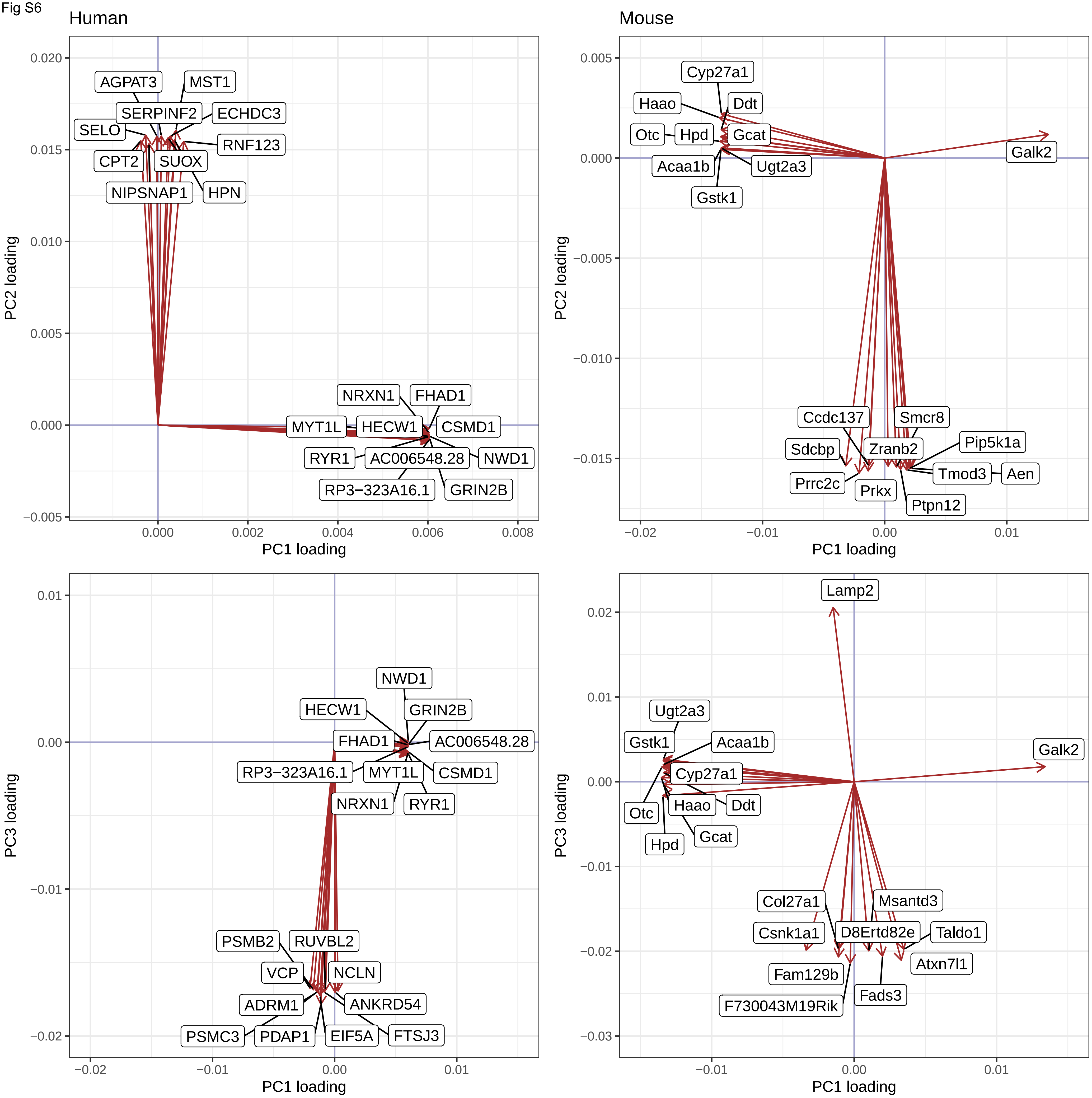
Human
Mouse
PC1 loading
PC2 loading
PC3 loading
AGPAT3
MST1
SERPINF2
ECHDC3
SELO
RNF123
CPT2
SUOX
NIPSNAP1
HPN
NRXN1
FHAD1
MYT1L
HECW1
CSMD1
RYR1
AC006548.28
NWD1
RP3−323A16.1
GRIN2B
Cyp27a1
Haao
Ddt
Otc
Hpd
Gcat
Acaa1b
Ugt2a3
Gstk1
Galk2
Ccdc137
Smcr8
Sdcbp
Zranb2
Pip5k1a
Tmod3
Aen
Prrc2c
Prkx
Ptpn12
PSMB2
RUVBL2
VCP
NCLN
ADRM1
ANKRD54
PSMC3
PDAP1
EIF5A
FTSJ3
Lamp2
Gstk1
Col27a1
Msantd3
Csnk1a1
D8Ertd82e
Taldo1
Atxn7l1
Fam129b
F730043M19Rik
Fads3

Supplemenatary Code

Setup

```
library(tidyverse)
library(ggplot2)
library(grid)
library(gridExtra)
library(SummarizedExperiment)
library(patchwork)
library(ggbeeswarm)

#loads an RData file, and returns it
loadRData <- function(fileName){
    load(fileName)
    get(ls()[ls() != "fileName"])
}

if (!dir.exists("plots")) dir.create("plots")

palette_categorical <- rep(RColorBrewer::brewer.pal(12, "Paired"), 4)

palette_cat_16 = c(
  "dodgerblue2",
  "#E31A1C",
  "green4",
  "#6A3D9A",
  "#FF7F00",
  "black",
  "gold1",
  "skyblue2",
  "palegreen2",
  "#FDBF6F",
  "gray70",
  "maroon",
  "orchid1",
  "darkturquoise",
  "darkorange4",
  "brown"
)
```

Load data

```
## Get pipeline output paths ---------------------------------------------------------------
cget_path <- params$cget_path
cgetd_path_mouse <- params$cgetd_path_mouse
cgetd_path_human <- params$cgetd_path_human
hitfile_path_human <- params$hitfile_path_human
hitfile_path_mouse <- params$hitfile_path_mouse

### Load sample metadata -------------------------------------------------------------------
sample_sheet_human <-
  read_tsv(paste0(cgetd_path_human, "pipelineSampleSheet.txt")) %>%
  mutate(sample_name = paste(incubation_medium, incubation_time_h,
                             donor_id, sep = "_"),
         Group = str_remove(Group, "Liver_PCLS_") %>% str_replace("_", " "))

sample_sheet_mouse <-
  read_tsv(paste0(cgetd_path_mouse, "pipelineSampleSheet.txt")) %>%
  mutate(sample_name = paste(treatment, timepoint, animal_id, sep = "_"),
         Group = str_remove(Group, "Liver_PCLS_") %>% str_replace("_", " "))

### Load RNASeq QC metrics -----------------------------------------------------------------
## If summary table not exists, read all individual per sample STAR metrics and aggregate
if (file.exists("input_files/rnaseq_qc_human.tsv")) {
  rnaseq_qc_human <- read_tsv("input_files/rnaseq_qc_human.tsv")
} else {
  rnaseq_qc_human <- sample_sheet_human$sampleId %>%
    map_df(~ read_tsv(
      paste0(
        cget_path,
        .x,
        "/",
        .x,
        "_star_genome_picardmetrics/",
        "collate-",
        .x,
        "_star_genome-all-metrics.tsv"
      )
    )) %>%
    mutate(sampleId = str_remove(SAMPLE, "_star_genome")) %>%
    left_join(sample_sheet_human %>% select(sampleId, sample_name)) %>%
    select(sampleId, sample_name, everything())

  stopifnot(all(sample_sheet_human$sampleId %in% rnaseq_qc_human$sampleId))
  write_tsv(rnaseq_qc_human, "input_files/rnaseq_qc_human.tsv")
}

if (file.exists("input_files/rnaseq_qc_mouse.tsv")) {
  rnaseq_qc_mouse <- read_tsv("input_files/rnaseq_qc_mouse.tsv")
} else {
  rnaseq_qc_mouse <- sample_sheet_mouse$sampleId %>%
    map_df(~ read_tsv(
      paste0(
        cget_path,
```

```r
        .x,
        "/",
        .x,
        "_star_genome_picardmetrics/",
        "collate-",
        .x,
        "_star_genome-all-metrics.tsv"
      )
    )) %>%
    mutate(sampleId = str_remove(SAMPLE, "_star_genome")) %>%
    left_join(sample_sheet_mouse %>% select(sampleId, sample_name)) %>%
    select(sampleId, sample_name, everything())

  stopifnot(all(sample_sheet_human$sampleId %in% rnaseq_qc_human$sampleId))
  write_tsv(rnaseq_qc_mouse, "input_files/rnaseq_qc_mouse.tsv")
}


### Load expression matrices -----------------------------------------------------------------
norm_counts_human <-
  loadRData(paste0(cgetd_path_human, "results/exprData/exprMatrix.Rdata")) %>%
  assay("normCounts")

# sum(apply(norm_counts_human, 1, var) == 0)
norm_counts_human <-
  norm_counts_human[which(apply(norm_counts_human, 1, var) != 0), ]
# 1268 genes have var == 0 and were removed

norm_counts_mouse <-
  loadRData(paste0(cgetd_path_mouse, "results/exprData/exprMatrix.Rdata")) %>%
  assay("normCounts")

# sum(apply(norm_counts_mouse, 1, var) == 0)
norm_counts_mouse <-
  norm_counts_mouse[which(apply(norm_counts_mouse, 1, var) != 0), ]
# 11352 genes have var == 0 and were removed


### Load Ensembl gene and orthologs info -------------------------------------------------
genes <-
  readr::read_tsv(
    "input_files/Ensembl_silicat_table.txt",
    col_types = cols(chr_name = "c")
  )

gene_info_h <- read_csv("input_files/gene_info_human.csv") %>%
  mutate(biotype2 = if_else(biotype == "protein_coding",
                            "Protein coding",
                            "Not protein coding"))

gene_info_m <- read_csv("input_files/gene_info_mouse.csv") %>%
  mutate(biotype2 = if_else(biotype == "protein_coding",
                            "Protein coding",
                            "Not protein coding"))
```

```r
orthologs_1to1 <- read_csv("input_files/orthologs_1to1.csv")
```

Figure 1. Intracellular triglyceride content of human samples

```
## Filter metadata of samples from 48h.
mdata_48h <- sample_sheet_human %>%
  filter(incubation_time_h == "48") %>%
  arrange(cget_id)

tg_levels <- mdata_48h %>%
  mutate(tg_after = as.numeric(na_if(tg_after, "not measured yet")),
         tg_delta = tg_after - tg_before,
         tg_delta_perc = ((tg_delta / tg_before) * 100) %>% round(2)) %>%
  arrange(sampleId) %>%
  column_to_rownames("sampleId") %>%
  select(tg_before, tg_after, tg_delta, tg_delta_perc)
```

```
plot_triglicerydes <- function(value, y_scale) {
  mdata_48h %>%
    select(sampleId, incubation_medium, donor_id) %>%
    left_join(tg_levels %>% rownames_to_column("sampleId")) %>%
    pivot_longer(cols = starts_with("tg_"),
                 names_to = "measurement_type",
                 values_to = "measurement_value") %>%
    mutate(measurement_type = factor(measurement_type,
                                     levels = c("tg_before",
                                                "tg_after",
                                                "tg_delta",
                                                "tg_delta_perc"))) %>%
    filter(measurement_type == value) %>%
    ggplot(aes(x = incubation_medium, y = measurement_value)) +
    geom_boxplot(outlier.alpha = 0,
                 width = 0.3,
                 position = position_nudge(-0.1)) +
    gghalves::geom_half_point_panel(aes(color = donor_id),
                              transformation = ggbeeswarm::position_quasirandom(
                                width = 0.1,
                                groupOnX = TRUE),
                              alpha = 0.5) +
    theme_bw() +
    facet_wrap(~measurement_type,
               nrow = 1,
               scales = if_else(y_scale == "free", "free_y", "fixed"),
               strip.position = "left",
               labeller = as_labeller(c(tg_after = "tg 48 h (ug/mg)",
                                        tg_before = "tg 0 h (ug/mg)",
                                        tg_delta = "tg delta (ug/mg)",
                                        tg_delta_perc = "tg delta (%)"))) +
    ylab(NULL) +
    theme(strip.background = element_blank(),
          strip.placement = "outside") +
    labs(color = "Donor") +
    theme(axis.title.x = element_blank()) +
    if (y_scale == "fixed") coord_cartesian(ylim = c(0, 160))
}
```

```r
p1 <- plot_triglicerydes("tg_before", y_scale = "fixed") +
  theme(axis.text.x = element_blank(),
        axis.ticks.x = element_blank())
p2 <- plot_triglicerydes("tg_after", y_scale = "fixed") +
  theme(axis.text.x = element_blank(),
        axis.ticks.x = element_blank())
p3 <- plot_triglicerydes("tg_delta", y_scale = "free")
p4 <- plot_triglicerydes("tg_delta_perc", y_scale = "free")

p1 + p2 + p3 + p4 + plot_layout(guides = "collect", ncol = 2)
```

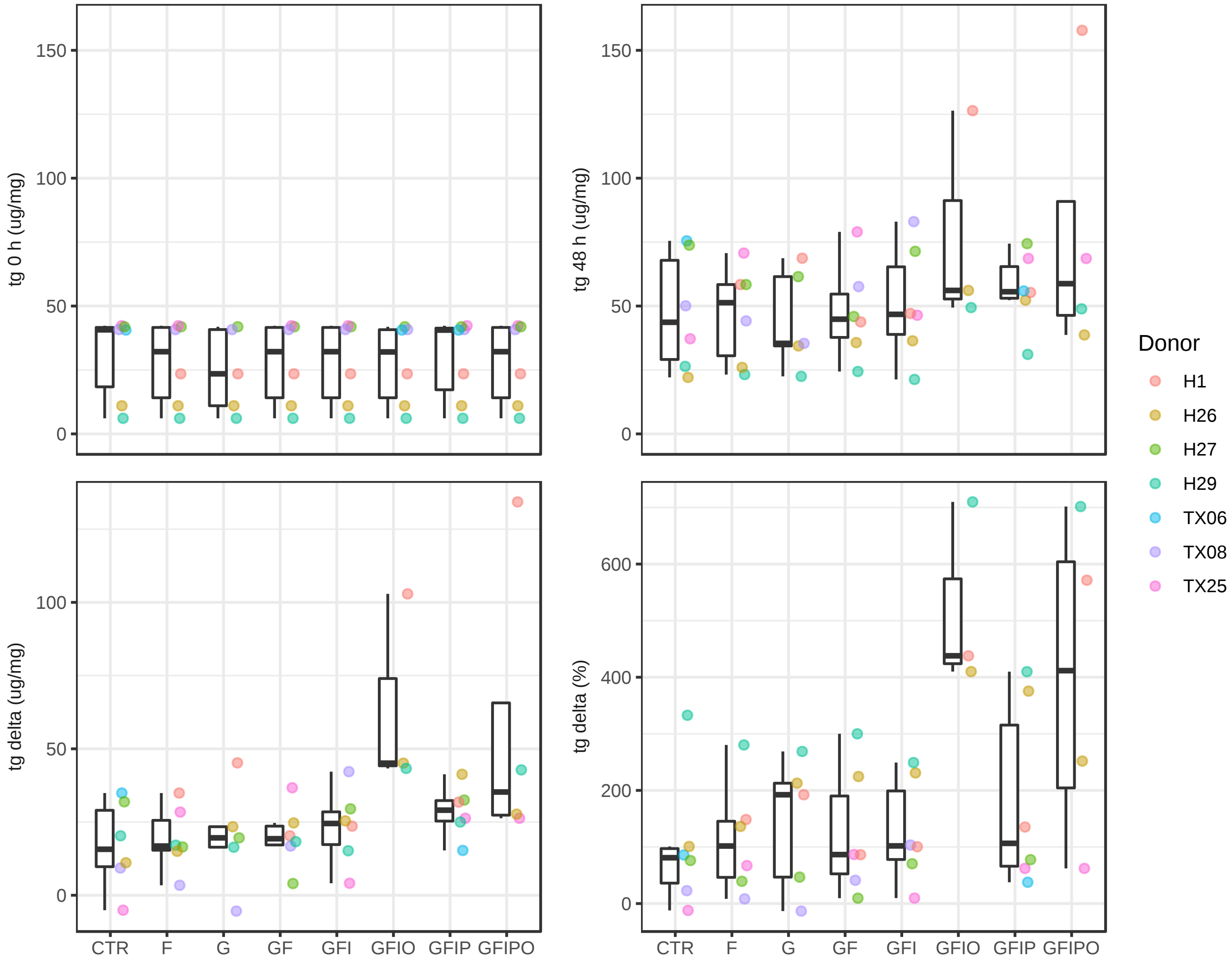


```r
ggsave("plots/Fig_1._Intracellular_triglyceride_content_of_human_samples.pdf",
       width = 9,
       height = 7)

ggsave("plots_png/Fig_1._Intracellular_triglyceride_content_of_human_samples.png",
       width = 9,
       height = 7)
```

Figure 2. Sequencing metrics from RNASeq data analysis

```
plot_reads_stacked_bar <- function(qc_metrics_df) {
  df_to_plot <- qc_metrics_df %>%
    mutate(
      pct_exonic_bases = (CODING_BASES + UTR_BASES) / PF_ALIGNED_BASES,
      est_num_exonic_reads = pct_exonic_bases * PF_HQ_ALIGNED_READS,
      "Pass filter" = PF_READS / 2 / 10^6,
      "Mapped" = PF_READS_ALIGNED / 2 / 10^6,
      "Uniquely mapped" = PF_HQ_ALIGNED_READS / 2 / 10^6,
      "Uniquely mapped to exons" = est_num_exonic_reads / 2 / 10^6) %>%
    select(
      sample_name,
      "Pass filter",
      "Mapped",
      "Uniquely mapped",
      "Uniquely mapped to exons"
    ) %>%
    pivot_longer(
      cols = c(
        "Pass filter",
        "Mapped",
        "Uniquely mapped",
        "Uniquely mapped to exons"
      ),
      names_to = "type"
    ) %>%
    mutate(type = factor(
      type,
      levels = c(
        "Pass filter",
        "Mapped",
        "Uniquely mapped",
        "Uniquely mapped to exons"
      )
    )) %>%
  separate(sample_name,
           into = c("condition", "timepoint", "donor_id"),
           sep = "_",
           remove = FALSE)

  df_to_plot %>%
    ggplot(aes(x = donor_id, y = value, fill = type)) +
    geom_bar(stat = "identity",
             position = "identity",
             alpha = 0.8,
             width = 1) +
    ylab("Millions of read pairs") +
    xlab("Sample") +
    theme_bw() +
    labs(fill = "Type") +
    ggh4x::facet_nested(~condition + timepoint,
                        scales = "free_x",
                        nest_line = TRUE,
```

```
                        switch = "x",
                        resect = unit(0.1, "in")
                        ) +
    theme(axis.text.x = element_text(angle = 60, hjust = 1),
          strip.background = element_blank(),
          strip.placement = "outside",
          panel.border = element_blank(),
          panel.background = element_blank(),
          panel.grid.major.x = element_blank(),
          panel.grid.major.y = element_line(linetype = "dashed", size = 0.3),
          panel.grid.minor.y = element_line(linetype = "dashed", size = 0.3),
          panel.ontop = TRUE
          ) +
    coord_cartesian(ylim = c(0, 90), expand = FALSE) +
    scale_y_continuous(breaks = scales::pretty_breaks(n = 11))
}
```

```
p1 <- plot_reads_stacked_bar(rnaseq_qc_human) +
  ggtitle("Human")

p2 <- plot_reads_stacked_bar(rnaseq_qc_mouse) +
  ggtitle("Mouse")

p1 / p2 + plot_layout(guides = "collect") &
  theme(legend.position = 'bottom')
```

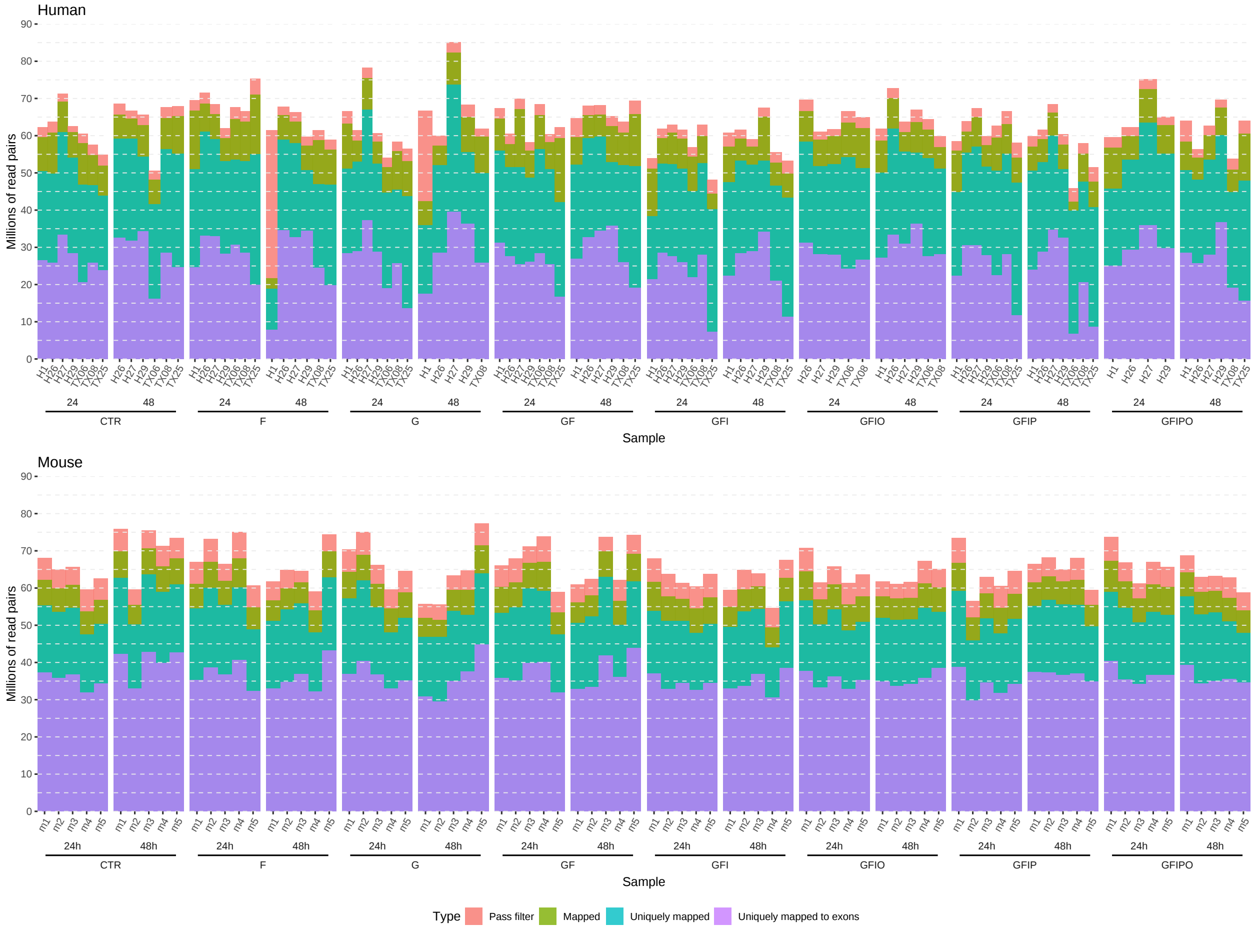


```
ggsave("plots/Fig_2._Sequencing_metrics_from_RNASeq_data_analysis.pdf",
       width = 16,
       height = 12)

ggsave("plots_png/Fig_2._Sequencing_metrics_from_RNASeq_data_analysis.png",
       width = 16,
       height = 12)
```

Figure 3. Principal Component Analysis

```r
## do PCA
pca_human <- prcomp(t(norm_counts_human), scale. = TRUE)
pca_mouse <- prcomp(t(norm_counts_mouse), scale. = TRUE)


## calculate % variance explained
hPcv <- (pca_human$sdev^2/sum(pca_human$sdev^2))*100
names(hPcv) <- paste0("PC", 1:length(hPcv))

mPcv <- (pca_mouse$sdev^2/sum(pca_mouse$sdev^2))*100
names(mPcv) <- paste0("PC", 1:length(mPcv))


## prepare PCA results for ggplot
hPca.df <-
  left_join(
    pca_human$x %>%
      as.data.frame() %>%
      rownames_to_column("sampleId"),
    sample_sheet_human %>%
      mutate(group = str_remove(Group, "Liver_PCLS_") %>%
               str_replace("_", " "),
             timepoint = ifelse(incubation_time_h == 24, "24 h", "48 h")) %>%
      select(sampleId, sample_name, group, incubation_medium, timepoint, donor_id)
  )

mPca.df <-
  left_join(
    pca_mouse$x %>%
      as.data.frame() %>%
      rownames_to_column("sampleId"),
    sample_sheet_mouse %>%
      mutate(group = str_remove(Group, "Liver_PCLS_") %>%
               str_replace("_", " "),
             timepoint = ifelse(timepoint == "24h", "24 h", "48 h")) %>%
      select(sampleId, sample_name, group, incubation_medium = treatment, timepoint,
             animal_id)
  )
```

```r
### PC1 vs PC2
hPc1.pc2 <- hPca.df %>%
  ggplot(aes(x = PC1, y = PC2, color = incubation_medium, shape = timepoint)) +
  geom_point(size = 2) +
  scale_shape_manual(values = 1:2) +
  ggrepel::geom_text_repel(aes(label = ifelse(PC1 > 200, sample_name, "")),
                           size = 3) +
  xlab(paste0("PC1 (", round(hPcv["PC1"], digits = 2), " %)")) +
  ylab(paste0("PC2 (", round(hPcv["PC2"], digits = 2), " %)")) +
  theme_bw() +
  ggtitle("Human") +
  labs(color = "Incubation medium", shape = "Incubation time") +
  coord_fixed(ratio = 1)
```

```
mPc1.pc2 <- mPca.df %>%
  ggplot(aes(x = PC1, y = PC2, color = incubation_medium, shape = timepoint)) +
  geom_point(size = 2) +
  scale_shape_manual(values = 1:2) +
  xlab(paste0("PC1 (", round(mPcv["PC1"], digits = 2), " %)")) +
  ylab(paste0("PC2 (", round(mPcv["PC2"], digits = 2), " %)")) +
  theme_bw() +
  ggtitle("Mouse") +
  labs(color = "Incubation medium", shape = "Incubation time") +
  coord_fixed(ratio = 1) +
  theme(legend.position = "none")
```

```
### PC1 vs PC3
hPc1.pc3 <- hPca.df %>%
  ggplot(aes(x = PC1, y = PC3, color = incubation_medium, shape = timepoint)) +
  geom_point(size = 2) +
  scale_shape_manual(values = 1:2) +
  ggrepel::geom_text_repel(aes(label = ifelse(PC1 > 200, sample_name, "")),
                           size = 3) +
  xlab(paste0("PC1 (", round(hPcv["PC1"], digits = 2), " %)")) +
  ylab(paste0("PC3 (", round(hPcv["PC3"], digits = 2), " %)")) +
  theme_bw() +
  ggtitle("Human") +
  labs(color = "Incubation medium", shape = "Incubation time") +
  coord_fixed(ratio = 1)

mPc1.pc3 <- mPca.df %>%
  ggplot(aes(x = PC1, y = PC3, color = incubation_medium, shape = timepoint)) +
  geom_point(size = 2) +
  scale_shape_manual(values = 1:2) +
  xlab(paste0("PC1 (", round(mPcv["PC1"], digits = 2), " %)")) +
  ylab(paste0("PC3 (", round(mPcv["PC3"], digits = 2), " %)")) +
  theme_bw() +
  ggtitle("Mouse") +
  labs(color = "Incubation medium", shape = "Incubation time") +
  coord_fixed(ratio = 1) +
  theme(legend.position = "none")
```

```
### PC2 vs PC3
hPc2.pc3 <- hPca.df %>%
  ggplot(aes(x = PC2, y = PC3, color = incubation_medium, shape = timepoint)) +
  geom_point(size = 2) +
  scale_shape_manual(values = 1:2) +
  ggrepel::geom_text_repel(aes(label = ifelse(PC3 > 200, sample_name, "")),
                           size = 3) +
  xlab(paste0("PC2 (", round(hPcv["PC2"], digits = 2), " %)")) +
  ylab(paste0("PC3 (", round(hPcv["PC3"], digits = 2), " %)")) +
  theme_bw() +
  ggtitle("Human") +
  labs(color = "Incubation medium", shape = "Incubation time") +
  coord_fixed(ratio = 1)

mPc2.pc3 <- mPca.df %>%
```

```r
  ggplot(aes(x = PC2, y = PC3, color = incubation_medium, shape = timepoint)) +
  geom_point(size = 2) +
  scale_shape_manual(values = 1:2) +
  xlab(paste0("PC2 (", round(mPcv["PC2"], digits = 2), " %)")) +
  ylab(paste0("PC3 (", round(mPcv["PC3"], digits = 2), " %)")) +
  theme_bw() +
  ggtitle("Mouse") +
  labs(color = "Incubation medium", shape = "Incubation time") +
  coord_fixed(ratio = 1) +
  theme(legend.position = "none")
```

```r
p12 <- hPc1.pc2 + mPc1.pc2
p13 <- hPc1.pc3 + mPc1.pc3
p23 <- hPc2.pc3 + mPc2.pc3

(p12 / p13 / p23) + plot_layout(guides = "collect")
```

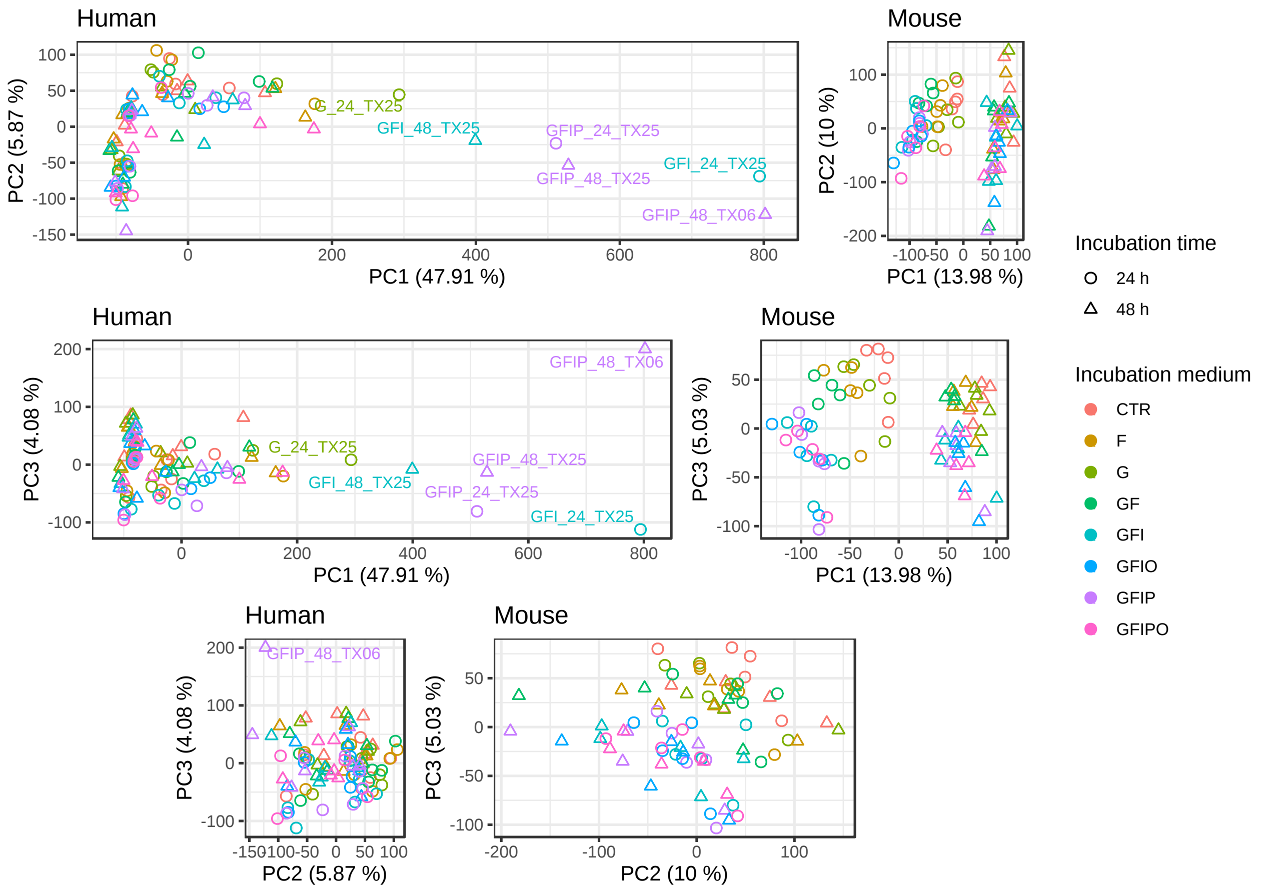


```r
ggsave("plots/Fig_3._Principal_Component_Analysis.pdf",
       width = 10,
       height = 7)

ggsave("plots_png/Fig_3._Principal_Component_Analysis.png",
       width = 10,
       height = 7)
```

Figure 4. Hierarchical clustering

```
## compute hierarchical clustering
hHc <- hclust(dist(t(norm_counts_human)))
hHc$labels <- data.frame(sampleId = hHc$labels) %>%
  left_join(sample_sheet_human) %>%
  pull(sample_name)

mHc <- hclust(dist(t(norm_counts_mouse)))
mHc$labels <- data.frame(sampleId = mHc$labels) %>%
  left_join(sample_sheet_mouse) %>%
  pull(sample_name)
```

```
## human dendrogram
p1 <- ggplotify::as.ggplot(~{
  timepoint_color <- ifelse(str_detect(hHc$labels, "24"), "#dddddd", "#999999")
  timepoint_map <- c("24 h" = "#dddddd", "48 h" = "#999999")

  group <-  hHc$labels %>% str_split("_") %>% map_chr(function(x) x[1])
  group_color <-  group %>% network::as.color()
  group_color <- RColorBrewer::brewer.pal(9, "Blues")[2:9][group_color]
  names(group_color) <- group
  group_map <- group_color[unique(group)]

  donor <- hHc$labels %>% str_split("_") %>% map_chr(function(x) x[3])
  donor_color <- donor %>% network::as.color()
  donor_color <- palette_cat_16[donor_color]
  names(donor_color) <- donor
  donor_map <- donor_color[unique(donor)]

  color_bars <- cbind(timepoint_color, group_color, donor_color)

  hHc$labels <- hHc$labels %>% str_replace_all("_", " ")

  par(mar = c(8.5, 1, 3, 0) + 0.1)

  dend_h <- hHc %>% as.dendrogram()

  plot(dend_h, horiz = FALSE, main = "Human")

  ## add color bar annotations
  dendextend::colored_bars(colors = color_bars,
                           dend = dend_h,
                           rowLabels = c("Timepoint", "Group", "Donor"),
                           horiz = FALSE)

  ## add legends
  legend(
    title = "Timepoint",
    x = "topright",
    legend = names(timepoint_map),
    pch = 15,
    pt.cex = 1.5,
    bty = 'n',
```

```
    x.intersp = 0.3,
    inset = c(0.14, 0.02), # place outside
    title.adj = 0.69,
    text.width = 1,
    col = timepoint_map
  )

  legend(
    title = "Donor",
    x = "topright",
    legend = names(donor_map),
    pch = 15,
    pt.cex = 1.5,
    bty = 'n',
    x.intersp = 0.3,
    inset = c(0.1, 0.02),
    title.adj = 0.61,
    text.width = 1,
    col = donor_map
  )

  legend(
    title = "Group",
    x = "topright",
    legend = names(group_map),
    pch = 15,
    pt.cex = 1.5,
    bty = 'n',
    x.intersp = 0.3,
    inset = c(0.05, 0.02),
    title.adj = 0.64,
    text.width = 1,
    col = group_map
  )
})


## mouse dendrogram
p2 <- ggplotify::as.ggplot(~{
  timepoint_color <- ifelse(str_detect(mHc$labels, "24"), "#dddddd", "#999999")
  timepoint_map <- c("24 h" = "#dddddd", "48 h" = "#999999")

  group <-  mHc$labels %>% str_split("_") %>% map_chr(function(x) x[1])
  group_color <-  group %>% network::as.color()
  group_color <- RColorBrewer::brewer.pal(9, "Blues")[2:9][group_color]
  names(group_color) <- group
  group_map <- group_color[unique(group)]

  donor <- mHc$labels %>% str_split("_") %>% map_chr(function(x) x[3])
  donor_color <- donor %>% network::as.color()
  donor_color <- palette_cat_16[donor_color]
  names(donor_color) <- donor
  donor_map <- donor_color[unique(donor)]
```

```r
  color_bars <- cbind(timepoint_color, group_color, donor_color)

  mHc$labels <- mHc$labels %>% str_replace_all("_", " ")

  par(mar = c(8.5, 1, 3, 0) + 0.1)

  dend_h <- mHc %>% as.dendrogram()

  plot(dend_h, horiz = FALSE, main = "Mouse")

  ## add color bar annotations
  dendextend::colored_bars(colors = color_bars,
                           dend = dend_h,
                           rowLabels = c("Timepoint", "Group", "Donor"),
                           horiz = FALSE)

  ## add legend
  legend(
    title = "Donor",
    x = "topright",
    legend = names(donor_map),
    pch = 15,
    pt.cex = 1.5,
    bty = 'n',
    x.intersp = 0.3,
    inset = c(0.05, 0.02),
    title.adj = 0.6,
    text.width = 1,
    col = donor_map
  )
})

p1 / p2
```

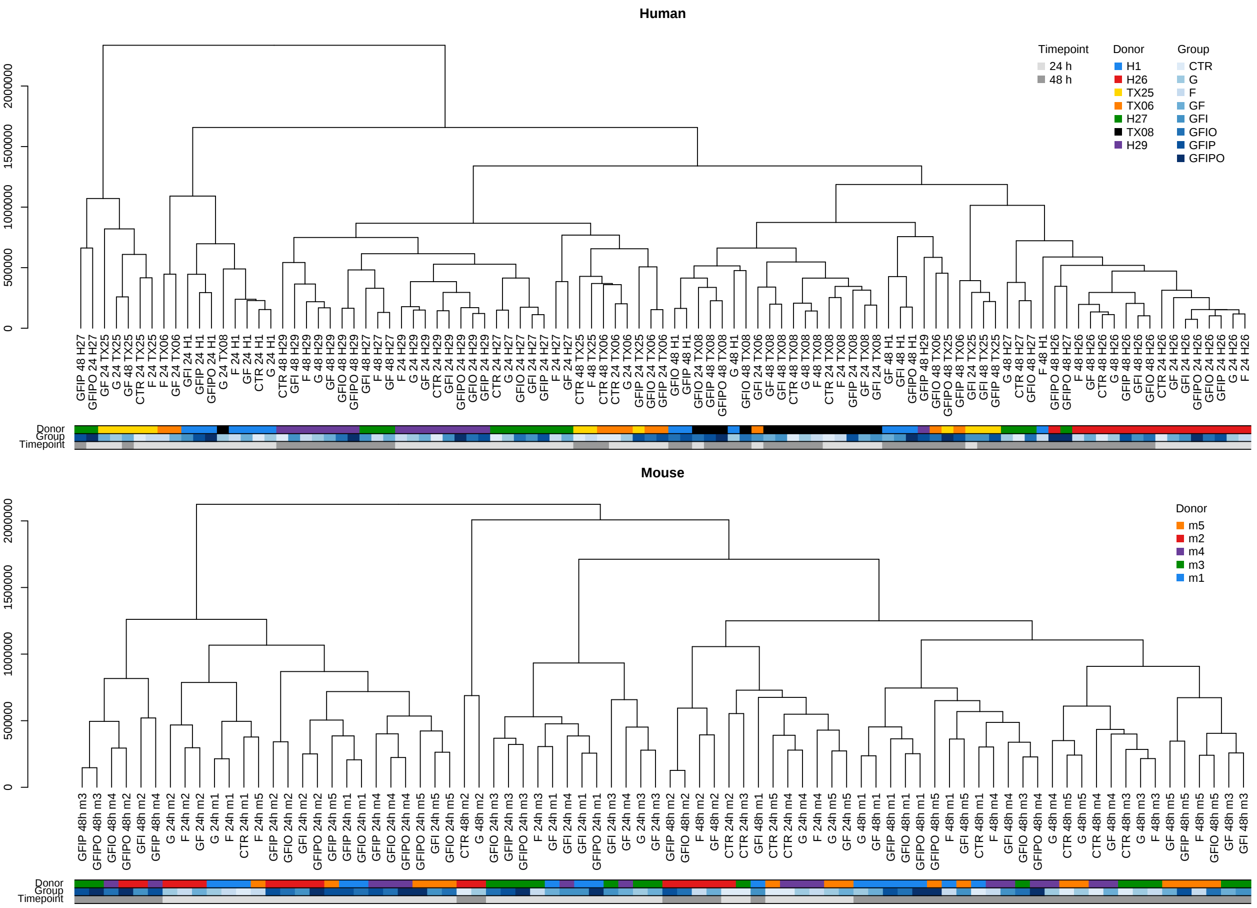


```r
ggsave("plots/Fig_4._Hierarchical_clustering.pdf",
       width = 20,
       height = 14)

ggsave("plots_png/Fig_4._Hierarchical_clustering.png",
       width = 20,
       height = 14)
```

Figure 5. Human vs. mouse gene expression correlation

```
set.seed(42)
orthologs_1to1_expressed <- intersect(
  intersect(rownames(norm_counts_human), orthologs_1to1$human_ensgid),
  filter(
    orthologs_1to1,
    mouse_ensgid %in% intersect(rownames(norm_counts_mouse),
                                orthologs_1to1$mouse_ensgid)
  ) %>%
    pull("human_ensgid")
)


med_normCounts_per_group_h <- sample_sheet_human %>%
  pull(Group) %>%
  unique() %>%
  set_names() %>%
  map_dfc(function(.x) {
    samples <- sample_sheet_human %>% filter(Group == .x) %>% pull(sampleId)
    norm_counts_human[, samples] %>%
      rowMedians()
  }) %>%
  mutate(ensgid = rownames(norm_counts_human)) %>%
  filter(ensgid %in% orthologs_1to1_expressed) %>%
  arrange(ensgid) %>%
  column_to_rownames("ensgid")


med_normCounts_per_group_m <- sample_sheet_human %>%
  pull(Group) %>%
  unique() %>%
  set_names() %>%
  map_dfc(function(.x) {
    samples <- sample_sheet_mouse %>% filter(Group == .x) %>% pull(sampleId)
    norm_counts_mouse[, samples] %>%
      rowMedians()
  }) %>%
  mutate(ensgid = rownames(norm_counts_mouse)) %>%
  inner_join(orthologs_1to1, by = c(ensgid = "mouse_ensgid")) %>%
  filter(human_ensgid %in% orthologs_1to1_expressed) %>%
  select(-ensgid, -global_identity_perc) %>%
  arrange(human_ensgid) %>%
  column_to_rownames("human_ensgid")

stopifnot(rownames(med_normCounts_per_group_h) == rownames(med_normCounts_per_group_m))

dfc <- full_join(
  med_normCounts_per_group_h %>%
    rownames_to_column("gene") %>%
    pivot_longer(cols = -gene, names_to = "group", values_to = "med_expr_human"),
  med_normCounts_per_group_m %>%
    rownames_to_column("gene") %>%
    pivot_longer(cols = -gene, names_to = "group", values_to = "med_expr_mouse"),
```

```r
  by = c("gene", "group")
) %>%
  filter(med_expr_human > 0, med_expr_mouse > 0)
```

```r
p1 <- dfc %>%
  ggplot(aes(x = med_expr_human, y = med_expr_mouse)) +
  geom_point(alpha = 0.2, shape = 1) +
  geom_smooth(method = "lm") +
  ggpubr::stat_cor(aes(x = med_expr_human, y = med_expr_mouse,
                       label = ..r.label..),
                       cor.coef.name = "R",
                   digits = 3, method = "pearson", color = "darkblue") +
  ggpubr::stat_cor(aes(x = med_expr_human, y = med_expr_mouse,
                       label = ..r.label..),
                       cor.coef.name = "rho",
                   digits = 3, method = "spearman", label.y.npc = 0.8) +
  facet_wrap(~ group) +
  theme_bw() +
  labs(x = "Median expression in human",
       y = "Median expression in mouse") +
  scale_y_log10(labels = scales::label_number_si()) +
  scale_x_log10(labels = scales::label_number_si()) +
  theme(strip.background = element_blank(),
        axis.text.x = element_text(angle = 60, hjust = 1))
```

```r
## Check how many genes are not found in the 1to1 orthologs table

# sum(!orthologs_1to1$human_ensgid %in% rownames(norm_counts_human)) # 324
# sum(!orthologs_1to1$mouse_ensgid %in% rownames(norm_counts_mouse)) # 1023
# sum(
#   !orthologs_1to1$human_ensgid %in% rownames(norm_counts_human) |
#   !orthologs_1to1$mouse_ensgid %in% rownames(norm_counts_mouse)
# ) # 1216

if (file.exists("input_files/orthologs_correlation.tsv")) {
  orthologs_expressed <- read_tsv("input_files/orthologs_correlation.tsv")
} else {
  ## get 1to1 orthologs between mouse and human
  orthologs_expressed <-
    orthologs_1to1 %>% filter(
      human_ensgid %in% rownames(norm_counts_human),
      mouse_ensgid %in% rownames(norm_counts_mouse)
    )

  orthologs_expressed <- orthologs_expressed %>% mutate(cor_pearson = NA_real_)

  # Iterate over genes
  for (row in 1:nrow(orthologs_expressed)) {
    human_gene <- orthologs_expressed$human_ensgid[row]
    mouse_gene <- orthologs_expressed$mouse_ensgid[row]

    ## Create vector of median per group expressions of current gene in human
    med_expr_h <- c()
    for (group in unique(sample_sheet_human$Group)) {
```

```r
      samples <- sample_sheet_human %>%
        filter(Group == group) %>%
        pull(sampleId)

      med_expr_h <- c(med_expr_h, median(norm_counts_human[human_gene, samples]))
    }

    ## Create vector of median per group expressions of current gene in mouse
    med_expr_m <- c()
    for (group in unique(sample_sheet_human$Group)) {
      samples <- sample_sheet_mouse %>%
        filter(Group == group) %>%
        pull(sampleId)
      med_expr_m <- c(med_expr_m, median(norm_counts_mouse[mouse_gene, samples]))
    }

    ## calculate pearson correlation between vectors of median per group
    ## expressions of current gene in human and mouse
    orthologs_expressed[row, "cor_pearson"] <-
      cor(med_expr_h, med_expr_m, method = "pearson")
  }
  write_tsv(orthologs_expressed, "input_files/orthologs_correlation.tsv")
}

## 1964 genes had standard deviation = 0 and thus correlation is NA
p2 <- ggplot(orthologs_expressed,
         aes(x=global_identity_perc,
             y=cor_pearson)) +
  geom_point(alpha = 0.1, size = 0.7) +
  theme_bw() +
  ylab("Pearson correlation coefficient") +
  xlab("Global sequence identity (%)") +
  coord_cartesian(xlim = c(0, 101), ylim = c(-1, 1), expand = FALSE)
```

```r
p1 + p2 +
  plot_annotation(tag_levels = 'A')
```

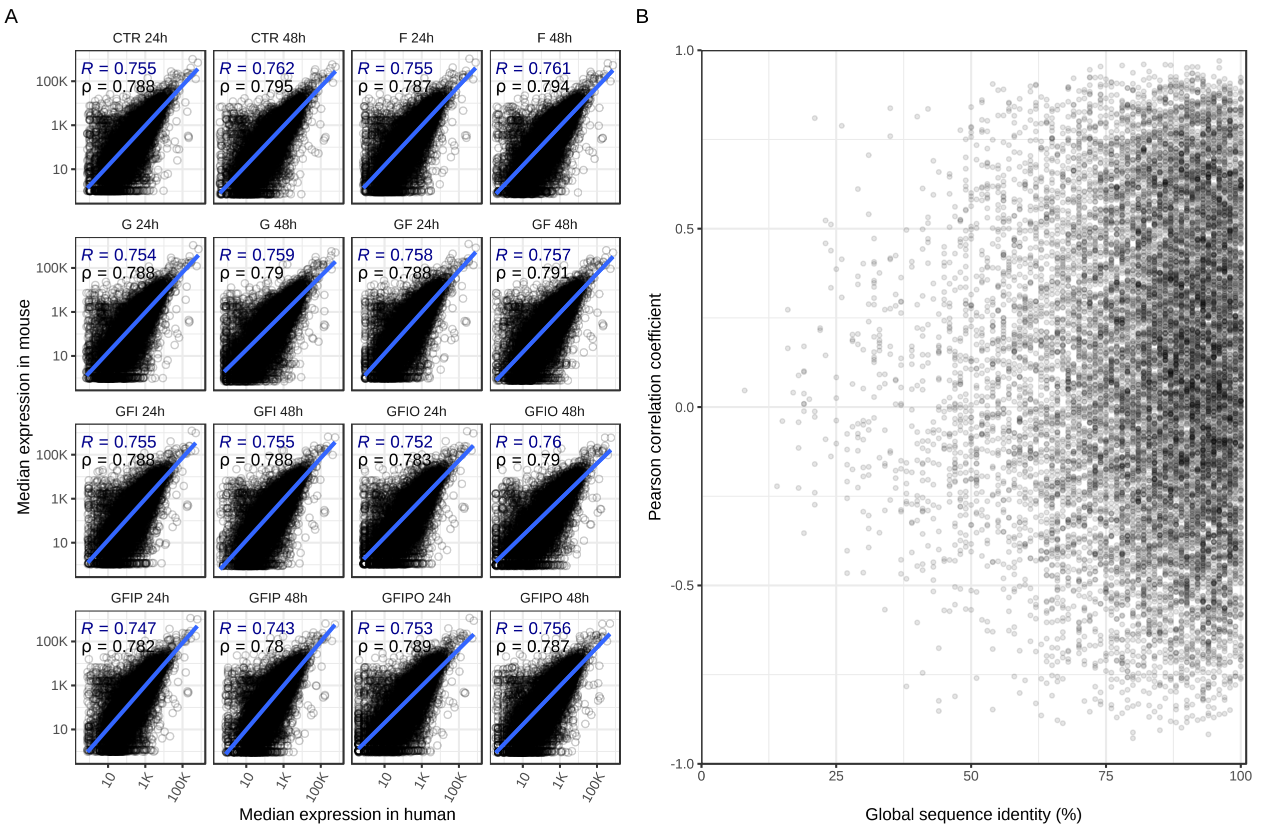


```
ggsave("plots/Fig_5._Human_vs._mouse_gene_expression_correlation.pdf",
       width = 12,
       height = 8)

ggsave("plots_png/Fig_5._Human_vs._mouse_gene_expression_correlation.png",
       width = 12,
       height = 8)
```

Figure 6. Top regulated genes based on experimental design

```r
## Read additional pipeline output containing various analyses of DEGs
conf_level <- "95"

hitfile_human <- paste(hitfile_path_human, conf_level,
                       "Confidence_results_human_proteins.txt", sep = "")

hitfile_mouse <- paste(hitfile_path_mouse, conf_level,
                       "Confidence_results_human_proteins.txt", sep = "")

hitdata_human <- read_tsv(hitfile_human, col_names = TRUE)
hitdata_mouse <- read_tsv(hitfile_mouse, col_names = TRUE)
```

```r
# Pairwise Comparisons
#  1 - G 24h vs. CTR 24h
#  2 - F 24h vs. CTR 24h
#  3 - GF 24h vs. CTR 24h
#  4 - GFI 24h vs. CTR 24h
#  5 - GFIO 24h vs. CTR 24h
#  6 - GFIP 24h vs. CTR 24h
#  7 - GFIPO 24h vs. CTR 24h
#  8 - G 48h vs. CTR 48h
#  9 - F 48h vs. CTR 48h
# 10 - GF 48h vs. CTR 48h
# 11 - GFI 48h vs. CTR 48h
# 12 - GFIO 48h vs. CTR 48h
# 13 - GFIP 48h vs. CTR 48h
# 14 - GFIPO 48h vs. CTR 48h
# 15 - CTR 48h vs. CTR 24h
# 16 - G 48h vs. G 24h
# 17 - F 48h vs. F 24h
# 18 - GF 48h vs. GF 24h
# 19 - GFI 48h vs. GFI 24h
# 20 - GFIO 48h vs. GFIO 24h
# 21 - GFIP 48h vs. GFIP 24h
# 22 - GFIPO 48h vs. GFIPO 24h

dh <- hitdata_human
dm <- hitdata_mouse
d <- dh %>% left_join(dm,
                      by = c("human_ensgid" = "human_ensgid...1"),
                      suffix = c(".h", ".m"))
## 4956 DEGs matched between human and mouse

d0 <- tibble(
  ensgid = d$human_ensgid,
  mouse_ensgid = d$homology_ensgid,
  flag_next_homolog = d$flag_next_homolog,
  flag_one2one = d$flag_one2one,
  gene_symbol = d$best_gene_name_ensembl.h,
  biotype = ifelse(d$target_class_prio.h > 0, "protein", "other"),

  # Human
```

```r
## For each DEG sum scores (logRatio * -log10padj) across contrasts
sum_score_h = d$`1_dge_score_Limma_model (CGET).h` +
  d$`2_dge_score_Limma_model (CGET).h` +
  d$`3_dge_score_Limma_model (CGET).h` +
  d$`4_dge_score_Limma_model (CGET).h` +
  d$`5_dge_score_Limma_model (CGET).h` +
  d$`6_dge_score_Limma_model (CGET).h` +
  d$`7_dge_score_Limma_model (CGET).h` +
  d$`8_dge_score_Limma_model (CGET).h` +
  d$`9_dge_score_Limma_model (CGET).h` +
  d$`10_dge_score_Limma_model (CGET).h` +
  d$`11_dge_score_Limma_model (CGET).h` +
  d$`12_dge_score_Limma_model (CGET).h`,

## For each DEG sum positive (+1) and negative (-1) signs of expression change
## across contrasts
sum_sign_h = d$`1_sign_Limma_model (CGET).h` +
  d$`2_sign_Limma_model (CGET).h` +
  d$`3_sign_Limma_model (CGET).h` +
  d$`4_sign_Limma_model (CGET).h` +
  d$`5_sign_Limma_model (CGET).h` +
  d$`6_sign_Limma_model (CGET).h` +
  d$`7_sign_Limma_model (CGET).h` +
  d$`8_sign_Limma_model (CGET).h` +
  d$`9_sign_Limma_model (CGET).h` +
  d$`10_sign_Limma_model (CGET).h` +
  d$`11_sign_Limma_model (CGET).h` +
  d$`12_sign_Limma_model (CGET).h`,

# Mouse
## For each DEG sum scores (logRatio * -log10padj) across contrasts
sum_score_m = d$`1_dge_score_Limma_model (CGET).m` +
  d$`2_dge_score_Limma_model (CGET).m` +
  d$`3_dge_score_Limma_model (CGET).m` +
  d$`4_dge_score_Limma_model (CGET).m` +
  d$`5_dge_score_Limma_model (CGET).m` +
  d$`6_dge_score_Limma_model (CGET).m` +
  d$`7_dge_score_Limma_model (CGET).m` +
  d$`8_dge_score_Limma_model (CGET).m` +
  d$`9_dge_score_Limma_model (CGET).m` +
  d$`10_dge_score_Limma_model (CGET).m` +
  d$`11_dge_score_Limma_model (CGET).m` +
  d$`12_dge_score_Limma_model (CGET).m`,

## For each DEG sum positive (+1) and negative (-1) signs of expression change
## across contrasts
sum_sign_m = d$`1_sign_Limma_model (CGET).m` +
  d$`2_sign_Limma_model (CGET).m` +
  d$`3_sign_Limma_model (CGET).m` +
  d$`4_sign_Limma_model (CGET).m` +
  d$`5_sign_Limma_model (CGET).m` +
  d$`6_sign_Limma_model (CGET).m` +
  d$`7_sign_Limma_model (CGET).m` +
```

```
    d$`8_sign_Limma_model (CGET).m` +
    d$`9_sign_Limma_model (CGET).m` +
    d$`10_sign_Limma_model (CGET).m` +
    d$`11_sign_Limma_model (CGET).m` +
    d$`12_sign_Limma_model (CGET).m`,

  # Sum signs across species
  sum_sign = sum_sign_h + sum_sign_m,
  # Sum scores across species
  sum_score = sum_score_h + sum_score_m,

  # For each DEG sum the absolute values of difference between the human and mouse sign.
  # Assigns: 0 if signs are the same, 2 if signs are opposite, 1 if the gene is shows
  # change in only one of the species.
  sum_diff_sign =
    abs(d$`1_sign_Limma_model (CGET).h` - d$`1_sign_Limma_model (CGET).m`) +
    abs(d$`2_sign_Limma_model (CGET).h` - d$`2_sign_Limma_model (CGET).m`) +
    abs(d$`3_sign_Limma_model (CGET).h` - d$`3_sign_Limma_model (CGET).m`) +
    abs(d$`4_sign_Limma_model (CGET).h` - d$`4_sign_Limma_model (CGET).m`) +
    abs(d$`5_sign_Limma_model (CGET).h` - d$`5_sign_Limma_model (CGET).m`) +
    abs(d$`6_sign_Limma_model (CGET).h` - d$`6_sign_Limma_model (CGET).m`) +
    abs(d$`7_sign_Limma_model (CGET).h` - d$`7_sign_Limma_model (CGET).m`) +
    abs(d$`8_sign_Limma_model (CGET).h` - d$`8_sign_Limma_model (CGET).m`) +
    abs(d$`9_sign_Limma_model (CGET).h` - d$`9_sign_Limma_model (CGET).m`) +
    abs(d$`10_sign_Limma_model (CGET).h` - d$`10_sign_Limma_model (CGET).m`) +
    abs(d$`11_sign_Limma_model (CGET).h` - d$`11_sign_Limma_model (CGET).m`) +
    abs(d$`12_sign_Limma_model (CGET).h` - d$`12_sign_Limma_model (CGET).m`)
  )

# filter by one2one mapping mouse genes
d1 <- d0 %>% filter(
  flag_one2one == 1 & biotype == "protein"
)

p1 <- ggplot(d1, aes(x = sum_sign_h)) +
  geom_bar() +
  xlab("Sum of signs across contrasts treated vs. CTR (human)") +
  ylab("N human genes") +
  theme_bw()

p2 <- ggplot(d1, aes(x = sum_sign)) +
  geom_bar() +
  xlab("Sum of signs across contrasts treated vs. CTR (human + mouse)") +
  ylab("N human and mouse genes") +
  theme_bw()

p3 <- ggplot(d1, aes(x = sum_diff_sign)) +
  geom_bar() +
  xlab("Sum of differences in sign across contrasts treated vs. CTR (human - mouse)") +
  ylab("N human and mouse genes") +
  theme_bw()
```

```r
p1 / p2 / p3 + plot_annotation(tag_levels = "A")
```

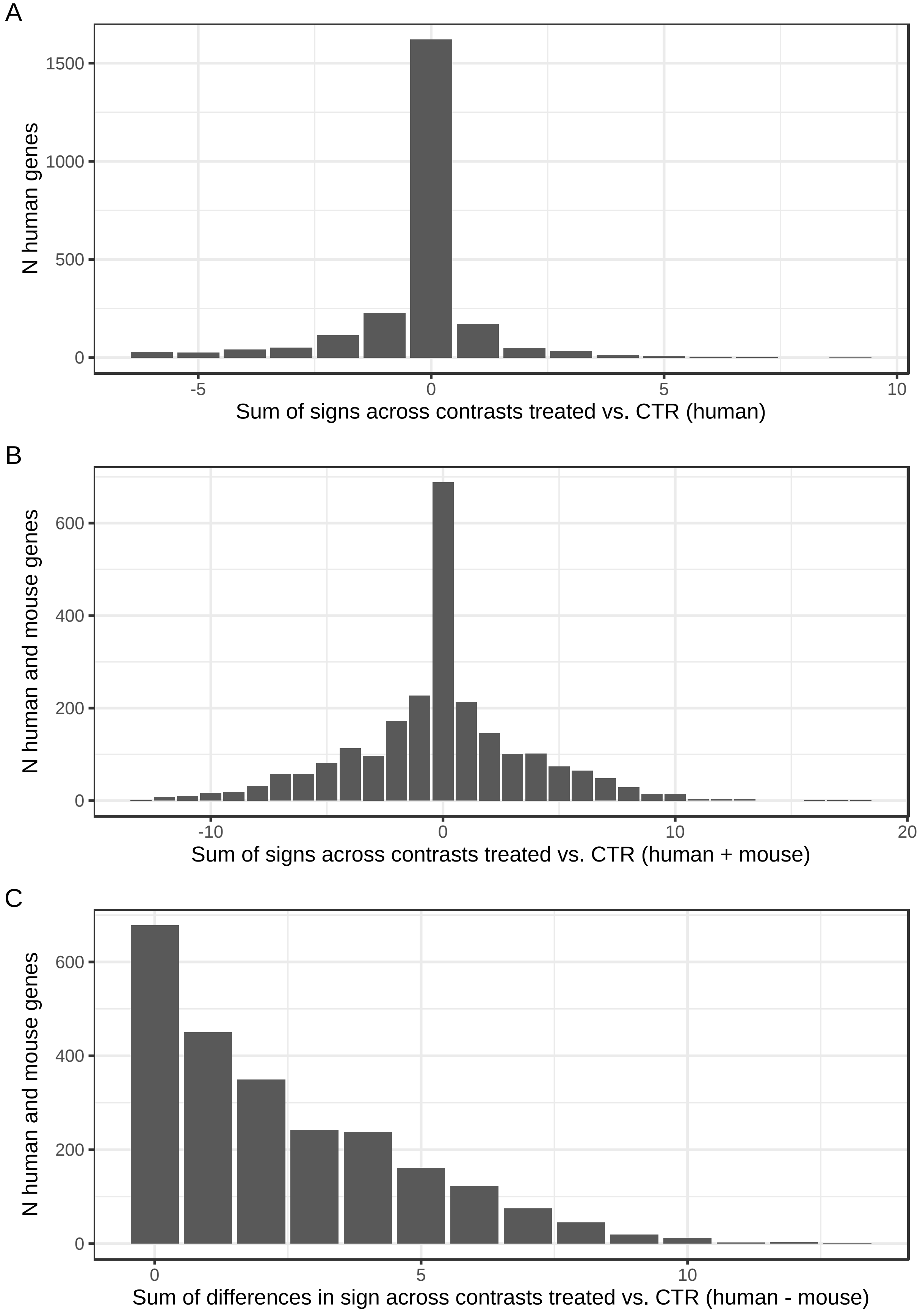
A
N human genes
1500
1000
500
0
-5
0
5
10
Sum of signs across contrasts treated vs. CTR (human)
B
N human and mouse genes
600
400
200
0
-10
0
10
20
Sum of signs across contrasts treated vs. CTR (human + mouse)
C
N human and mouse genes
600
400
200
0
0
5
10
Sum of differences in sign across contrasts treated vs. CTR (human - mouse)

```
ggsave("plots/Fig_6._Top_regulated_genes_based_on_experimental_design.pdf",
       width = 7,
       height = 10)

ggsave("plots_png/Fig_6._Top_regulated_genes_based_on_experimental_design.png",
       width = 7,
       height = 10)
```

Figure 7. Expression pattern of top correlated genes

```
tpm_human <-
  loadRData(paste0(cgetd_path_human, "results/exprData/exprMatrix.Rdata")) %>%
  assay("tpm")

tpm_mouse <-
  loadRData(paste0(cgetd_path_mouse, "results/exprData/exprMatrix.Rdata")) %>%
  assay("tpm")

cget_mapping_df <- bind_rows(
  sample_sheet_human %>%
    mutate(timepoint = if_else(incubation_time_h == "24", "24 h", "48 h")) %>%
    select(cget_id = sampleId, Group, donor_id, timepoint),
  sample_sheet_mouse %>%
    mutate(timepoint = if_else(timepoint == "24h", "24 h", "48 h")) %>%
    select(cget_id = sampleId, Group, donor_id = animal_id, timepoint)
)
```

```
plot_single_gene_expr <- function(human_ens_id, mouse_ens_id, gene_name) {
  expression_df <- bind_rows(
    data.frame(
      tpm = tpm_human %>%
        magrittr::extract(human_ens_id, ),
      species = "human"
    ),
    data.frame(
      tpm = tpm_mouse %>%
        magrittr::extract(mouse_ens_id, ),
      species = "mouse"
    )
  ) %>%
    rownames_to_column("cget_id") %>%
    left_join(cget_mapping_df, by = "cget_id") %>%
    mutate(Group = factor(Group, levels = c(
  "CTR 24h",
  "F 24h",
  "G 24h",
  "GF 24h",
  "GFI 24h",
  "GFIO 24h",
  "GFIP 24h",
  "GFIPO 24h",

  "CTR 48h",
  "F 48h",
  "G 48h",
  "GF 48h",
  "GFI 48h",
  "GFIO 48h",
  "GFIP 48h",
  "GFIPO 48h"
    )))
```

```
  expression_df %>%
    ggplot(aes(y = Group, x = tpm)) +
    geom_boxplot(aes(color = timepoint)) +
    scale_y_discrete(limits = rev,
                     labels = ~str_remove(., "24h|48h")) +
    facet_wrap(~species,
               labeller = as_labeller(c(human = "Human",
                                        mouse = "Mouse"))) +
    theme_bw() +
    xlab("TPM") +
    labs(color = "Timepoint") +
    ggtitle(gene_name) +
    theme(axis.title.y = element_blank()) +
    theme(strip.background = element_blank()) +
    scale_x_continuous(breaks = scales::breaks_extended(n = 8))
}
```

```
p1 <- plot_single_gene_expr(
  human_ens_id = "ENSG00000166147",
  mouse_ens_id = "ENSMUSG00000027204",
  gene_name = "FBN1"
)

p2 <- plot_single_gene_expr(
  human_ens_id = "ENSG00000168542",
  mouse_ens_id = "ENSMUSG00000026043",
  gene_name = "COL3A1"
)

p1 + p2 + plot_layout(guides = "collect") + plot_annotation(tag_levels = "A")
```

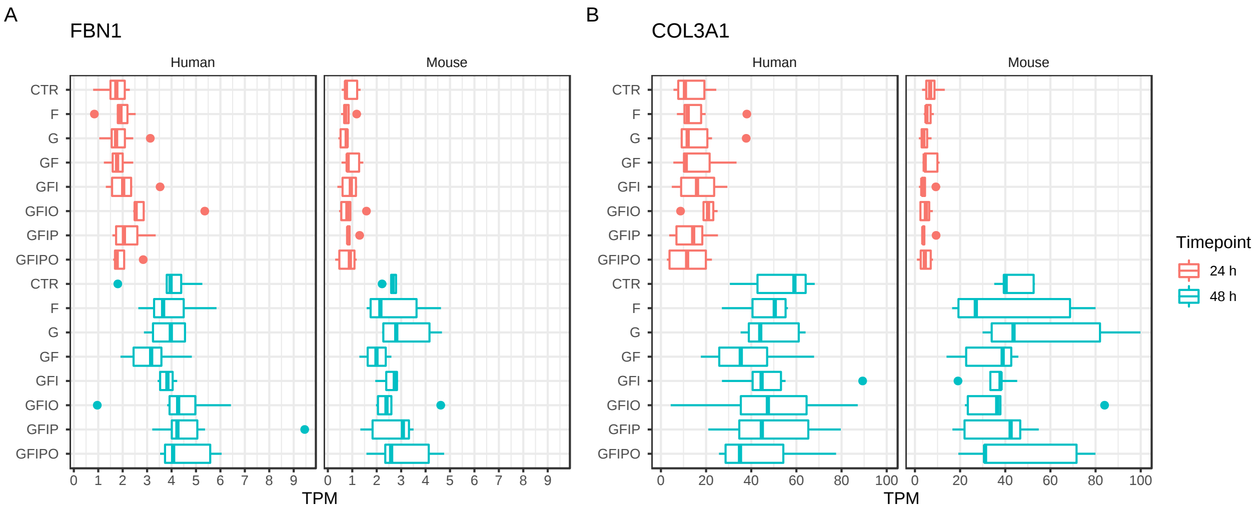


```
ggsave("plots/Fig_7._Expression_pattern_of_top_correlated_genes.pdf",
       width = 12,
       height = 5)

ggsave("plots_png/Fig_7._Expression_pattern_of_top_correlated_genes.png",
       width = 12,
       height = 5)
```

Figure 8. Expression pattern of top regulated genes

```
p1 <- plot_single_gene_expr(
  human_ens_id = "ENSG00000134363",
  mouse_ens_id = "ENSMUSG00000021765",
  gene_name = "FST"
)

p2 <- plot_single_gene_expr(
  human_ens_id = "ENSG00000140450",
  mouse_ens_id = "ENSMUSG00000042659",
  gene_name = "ARRDC4"
)

p1 + p2 + plot_layout(guides = "collect") + plot_annotation(tag_levels = "A")
```

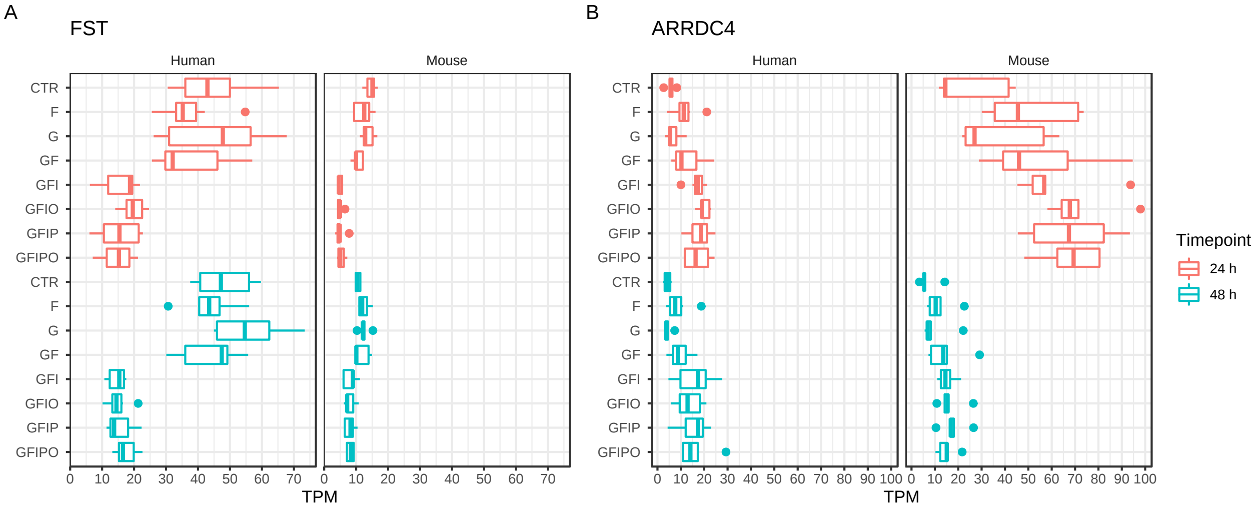


```
ggsave("plots/Fig_8._Expression_pattern_of_top_regulated_genes.pdf",
       width = 12,
       height = 5)

ggsave("plots_png/Fig_8._Expression_pattern_of_top_regulated_genes.png",
       width = 12,
       height = 5)
```

Figure 9. Expression pattern of top genes with species differences

```
p1 <- plot_single_gene_expr(
  human_ens_id = "ENSG00000135821",
  mouse_ens_id = "ENSMUSG00000026473",
  gene_name = "GLUL"
)

p2 <- plot_single_gene_expr(
  human_ens_id = "ENSG00000185567",
  mouse_ens_id = "ENSMUSG00000072812",
  gene_name = "AHNAK2"
)

p1 + p2 + plot_layout(guides = "collect") + plot_annotation(tag_levels = "A")
```

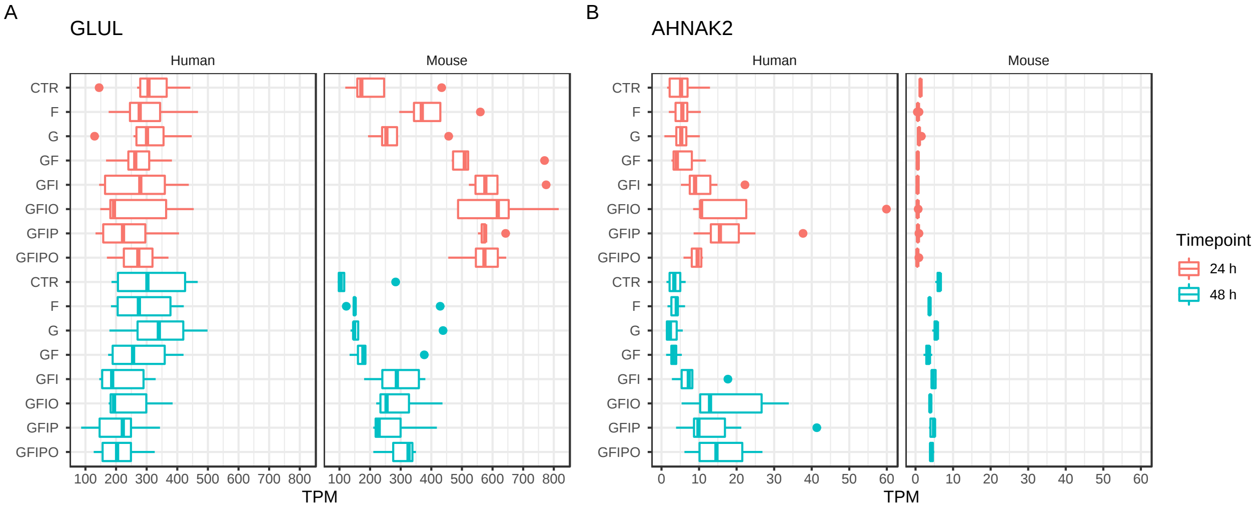


```
ggsave("plots/Fig_9._Expression_pattern_of_top_genes_with_species_differences.pdf",
       width = 12,
       height = 5)

ggsave("plots_png/Fig_9._Expression_pattern_of_top_genes_with_species_differences.png",
       width = 12,
       height = 5)
```

Figure 10. Expression pattern of selected ECM biomarker genes

```
p1 <- plot_single_gene_expr(
  human_ens_id = "ENSG00000107796",
  mouse_ens_id = "ENSMUSG00000035783",
  gene_name = "ACTA2"
)

p2 <- plot_single_gene_expr(
  human_ens_id = "ENSG00000108821",
  mouse_ens_id = "ENSMUSG00000001506",
  gene_name = "COL1A1"
)

p3 <- plot_single_gene_expr(
  human_ens_id = "ENSG00000115414",
  mouse_ens_id = "ENSMUSG00000026193",
  gene_name = "FN1"
)

p4 <- plot_single_gene_expr(
  human_ens_id = "ENSG00000149257",
  mouse_ens_id = "ENSMUSG00000070436",
  gene_name = "SERPINH1"
)

p1 + p2 + p3 + p4 + plot_layout(guides = "collect") + plot_annotation(tag_levels = "A")
```

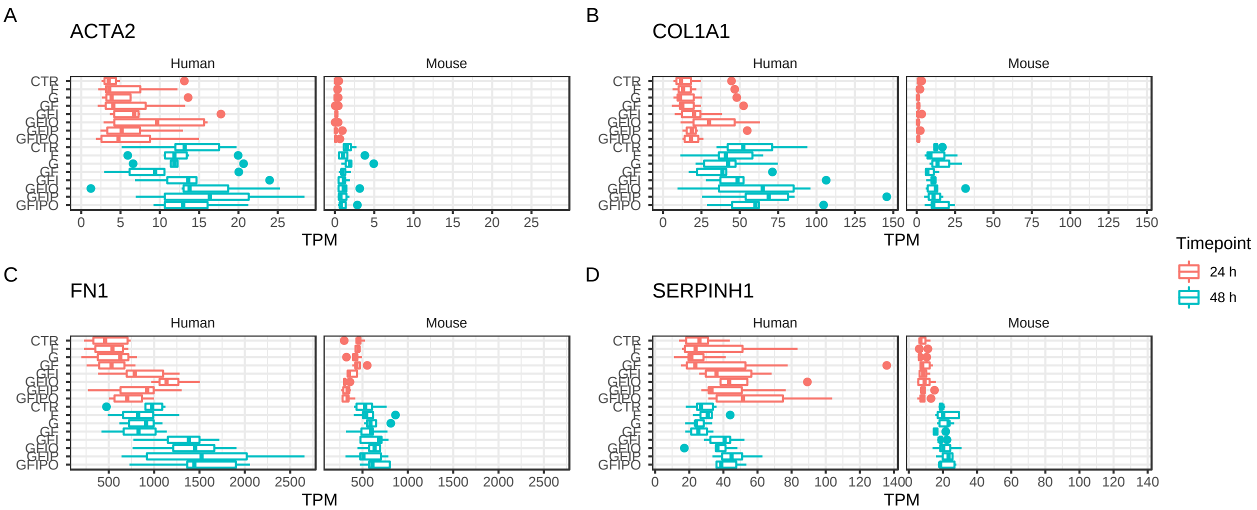


```
ggsave("plots/Fig_10._Expression_pattern_of_selected_ECM_biomarker_genes.pdf",
       width = 12,
       height = 10)

ggsave("plots_png/Fig_10._Expression_pattern_of_selected_ECM_biomarker_genes.png",
       width = 12,
       height = 10)
```

Supplemental Figure S1. RNA quality and yield

```
p1 <- sample_sheet_human %>%
  ggplot(aes(x = Group, y = rin)) +
  geom_boxplot() +
  geom_beeswarm(aes(color = donor_id)) +
  geom_hline(yintercept = 7.5, color = "darkgray", linetype = "longdash") +
  ggtitle("Human") +
  ylab("RIN") +
  theme_bw() +
  theme(axis.text.x = element_text(angle = 60, hjust = 1),
        panel.grid.minor.y = element_blank()) +
  coord_cartesian(ylim = c(0, 10)) +
  scale_y_continuous(breaks = scales::breaks_extended(n = 11)) +
  labs(color = "Human donor") +
  theme(axis.title.x = element_blank(),
        axis.text.x = element_blank())
```

```
p2 <- sample_sheet_mouse %>%
  ggplot(aes(x = Group, y = rin)) +
  geom_boxplot(outlier.size = 0) +
  geom_beeswarm(aes(color = animal_id)) +
  geom_hline(yintercept = 7.5, color = "darkgray", linetype = "longdash") +
  ggtitle("Mouse") +
  ylab("RIN") +
  theme_bw() +
  theme(axis.text.x = element_text(angle = 60, hjust = 1),
        panel.grid.minor.y = element_blank()) +
  coord_cartesian(ylim = c(0, 10)) +
  scale_y_continuous(breaks = scales::breaks_extended(n = 11)) +
  labs(color = "Mouse donor") +
  theme(axis.title.y = element_blank(),
        axis.title.x = element_blank(),
        axis.text.x = element_blank())
```

```
p3 <- sample_sheet_human %>%
  ggplot(aes(x = Group, y = rna_concentration)) +
  geom_boxplot() +
  geom_beeswarm(aes(color = donor_id)) +
  ylab("RNA yield [ng/uL]") +
  theme_bw() +
  theme(axis.text.x = element_text(angle = 60, hjust = 1),
        panel.grid.minor.y = element_blank()) +
  scale_y_continuous(breaks = scales::breaks_extended(n = 11),
                     limits = c(0, 250)) +
  labs(color = "Human donor") +
  theme(axis.title.x = element_blank())
```

```
p4 <- sample_sheet_mouse %>%
  ggplot(aes(x = Group, y = rna_conc)) +
  geom_boxplot() +
  geom_beeswarm(aes(color = animal_id)) +
  ylab("RNA yield [ng/uL]") +
  theme_bw() +
  theme(axis.text.x = element_text(angle = 60, hjust = 1),
```

```
       panel.grid.minor.y = element_blank()) +
  scale_y_continuous(breaks = scales::breaks_extended(n = 11),
                     limits = c(0, 250)) +
  labs(color = "Mouse donor") +
  theme(axis.title.x = element_blank(),
        axis.title.y = element_blank())
```

```
(p1 + p2) / (p3 + p4) + plot_layout(guides = "collect")
```

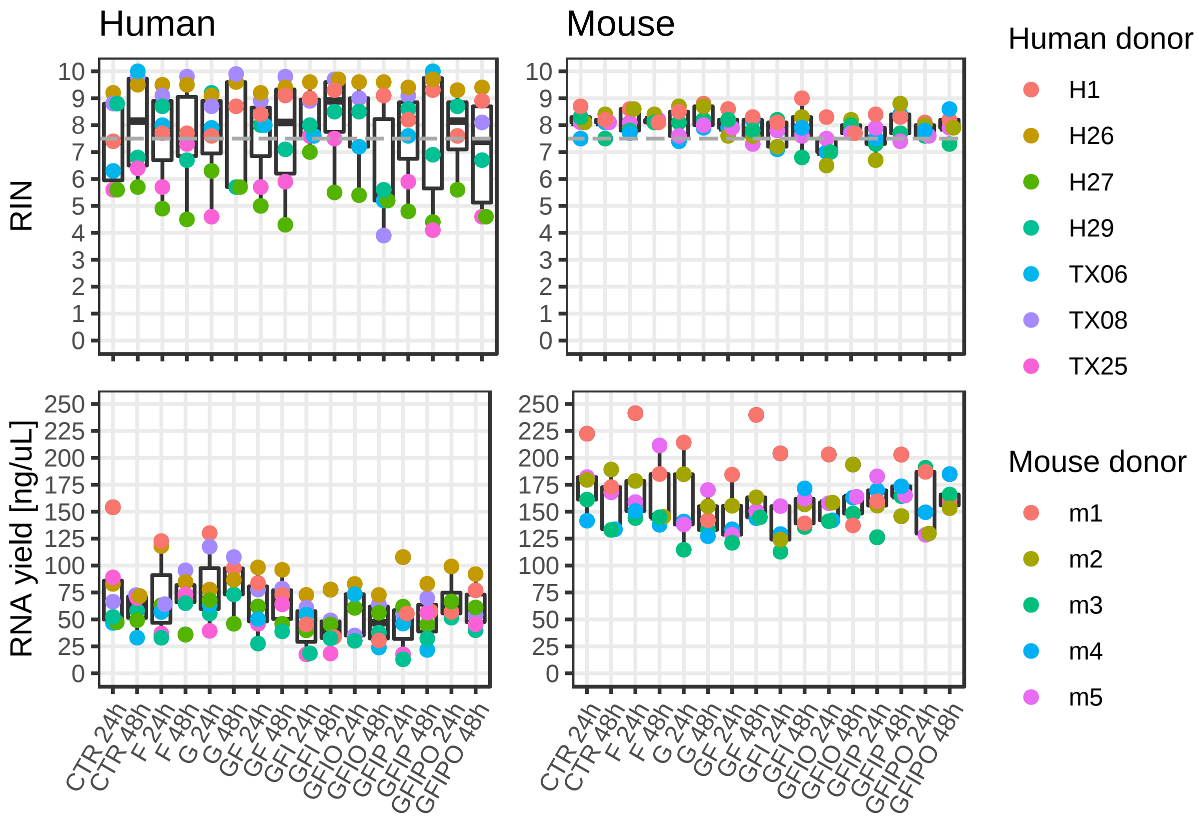


```
ggsave("plots/Supp_Fig_S1._RNA_quality_and_yield.pdf")

ggsave("plots_png/Supp_Fig_S1._RNA_quality_and_yield.png")
```

Supplemental Figure S2. Gene Biotype

```
## Count genes per group excluding those that have normCount = 0 in all samples belonging
## to the group.

detected_genes_h <- map(
  .x = unique(sample_sheet_human$Group),
  .f = function(.x) {
    samples <- sample_sheet_human %>%
      filter(Group == .x) %>%
      pull(sampleId)

    expr_subset_group <- norm_counts_human[ , samples]
    expr_subset_group[rowSums(expr_subset_group) > 0, ] %>%
      rownames()
  }
)
names(detected_genes_h) <- unique(sample_sheet_human$Group)

detected_genes_m <- map(
  .x = unique(sample_sheet_human$Group),
  .f = function(.x) {
    samples <- sample_sheet_mouse %>%
      filter(Group == .x) %>%
      pull(sampleId)

    expr_subset_group <- norm_counts_mouse[ , samples]
    expr_subset_group[rowSums(expr_subset_group) > 0, ] %>%
      rownames()
  }
)
names(detected_genes_m) <- unique(sample_sheet_human$Group)
```

```
## count genes by biotype
detected_genes_biotype_h <- detected_genes_h %>%
  imap_dfr(
    ~ tibble(ensgid = .x) %>%
      left_join(gene_info_h, by = "ensgid") %>%
      dplyr::count(biotype) %>%
      mutate(Group = .y)
  )

detected_genes_biotype_m <- detected_genes_m %>%
  imap_dfr(
    ~ tibble(ensgid = .x) %>%
      left_join(gene_info_m, by = "ensgid") %>%
      dplyr::count(biotype) %>%
      mutate(Group = .y)
  )
```

```
p1 <- detected_genes_biotype_h %>%
  ggplot(aes(x = Group, y = n, fill = fct_reorder(biotype, n, .desc = TRUE))) +
  geom_bar(stat = "identity", position = "stack") +
  scale_fill_manual(values = palette_categorical,
```

```
                    name = "Type (ordered by decreasing proportion)") +
  theme_bw() +
  theme(axis.text.x = element_text(angle = 60, hjust = 1)) +
  ylab("Number of genes") +
  ggtitle("Human") +
  theme(axis.title.x = element_blank()) +
  scale_y_continuous(breaks = scales::breaks_extended(n = 6),
                     limits = c(0, 60000)) +
  coord_cartesian(expand = FALSE)

p2 <- detected_genes_biotype_m %>%
  ggplot(aes(x = Group, y = n, fill = fct_reorder(biotype, n, .desc = TRUE))) +
  geom_bar(stat = "identity", position = "stack") +
  theme_bw() +
  theme(axis.text.x = element_text(angle = 60, hjust = 1)) +
  scale_fill_manual(values = palette_categorical,
                    name = "Type (ordered by decreasing proportion)") +
  ylab("Number of genes") +
  ggtitle("Mouse") +
  theme(axis.title.x = element_blank()) +
  scale_y_continuous(breaks = scales::breaks_extended(n = 6),
                     limits = c(0, 30000)) +
  coord_cartesian(expand = FALSE)

p1 / p2
```

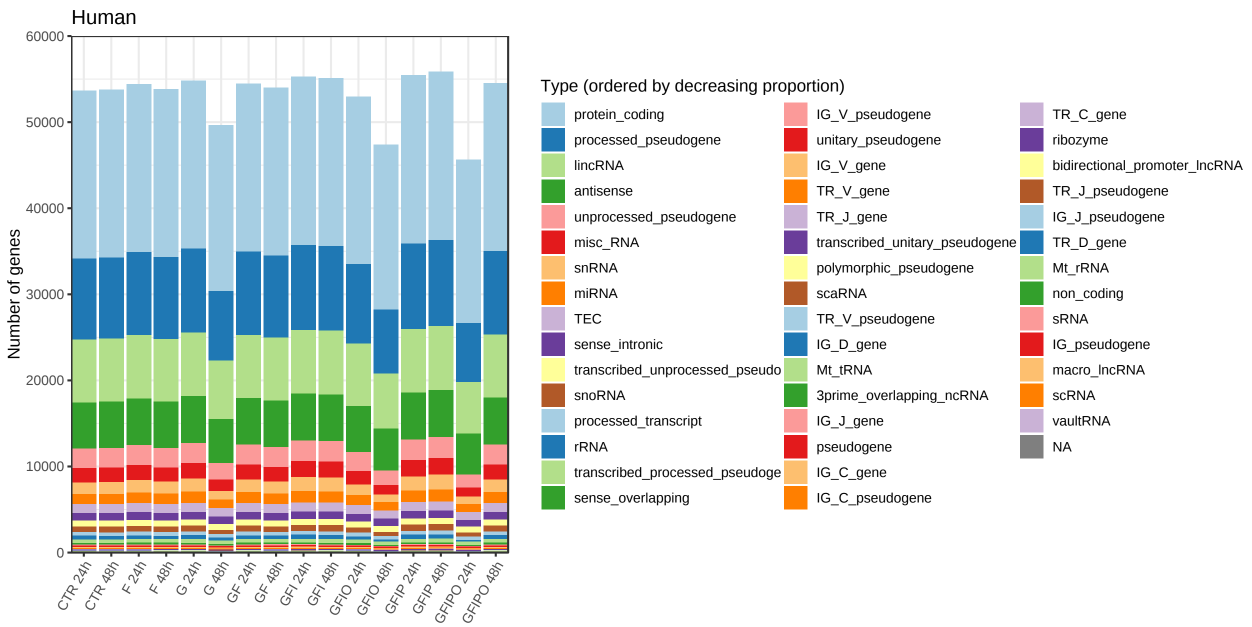

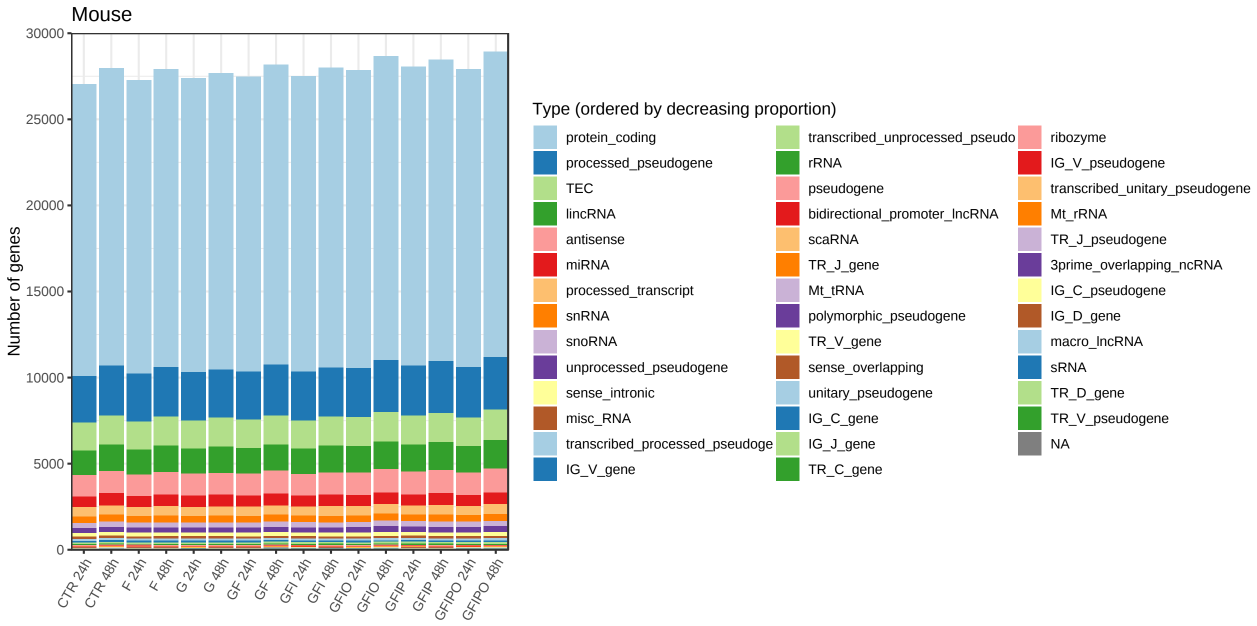


```
ggsave("plots/Supp_Fig_S2._Gene_Biotype.pdf",
       width = 12,
       height = 12)

ggsave("plots_png/Supp_Fig_S2._Gene_Biotype.png",
       width = 12,
       height = 12)
```

Supplemental Figure S3. Principal Component Analysis

```
### PC1 vs PC2
hPc1.pc2 <- hPca.df %>%
  ggplot(aes(x = PC1, y = PC2, color = donor_id, shape = timepoint)) +
  geom_point(size = 2) +
  scale_shape_manual(values = 1:2) +
  ggrepel::geom_text_repel(aes(label = ifelse(PC1 > 200, sample_name, "")),
                           size = 3) +
  xlab(paste0("PC1 (", round(hPcv["PC1"], digits = 2), " %)")) +
  ylab(paste0("PC2 (", round(hPcv["PC2"], digits = 2), " %)")) +
  theme_bw() +
  ggtitle("Human") +
  labs(color = "Human donor", shape = "Incubation time") +
  coord_fixed(ratio = 1)

mPc1.pc2 <- mPca.df %>%
  ggplot(aes(x = PC1, y = PC2, color = animal_id, shape = timepoint)) +
  geom_point(size = 2) +
  scale_shape_manual(values = 1:2) +
  xlab(paste0("PC1 (", round(mPcv["PC1"], digits = 2), " %)")) +
  ylab(paste0("PC2 (", round(mPcv["PC2"], digits = 2), " %)")) +
  theme_bw() +
  ggtitle("Mouse") +
  labs(color = "Mouse donor", shape = "Incubation time") +
  coord_fixed(ratio = 1)
```

```
### PC1 vs PC3
hPc1.pc3 <- hPca.df %>%
  ggplot(aes(x = PC1, y = PC3, color = donor_id, shape = timepoint)) +
  geom_point(size = 2) +
  scale_shape_manual(values = 1:2) +
  ggrepel::geom_text_repel(aes(label = ifelse(PC1 > 200, sample_name, "")),
                           size = 3) +
  xlab(paste0("PC1 (", round(hPcv["PC1"], digits = 2), " %)")) +
  ylab(paste0("PC3 (", round(hPcv["PC3"], digits = 2), " %)")) +
  theme_bw() +
  ggtitle("Human") +
  labs(color = "Human donor", shape = "Incubation time") +
  coord_fixed(ratio = 1)

mPc1.pc3 <- mPca.df %>%
  ggplot(aes(x = PC1, y = PC3, color = animal_id, shape = timepoint)) +
  geom_point(size = 2) +
  scale_shape_manual(values = 1:2) +
  xlab(paste0("PC1 (", round(mPcv["PC1"], digits = 2), " %)")) +
  ylab(paste0("PC3 (", round(mPcv["PC3"], digits = 2), " %)")) +
  theme_bw() +
  ggtitle("Mouse") +
  labs(color = "Mouse donor", shape = "Incubation time") +
  coord_fixed(ratio = 1)
```

```
### PC2 vs PC3
hPc2.pc3 <- hPca.df %>%
  ggplot(aes(x = PC2, y = PC3, color = donor_id, shape = timepoint)) +
```

```r
  geom_point(size = 2) +
  scale_shape_manual(values = 1:2) +
  ggrepel::geom_text_repel(aes(label = ifelse(PC3 > 200, sample_name, "")),
                           size = 3) +
  xlab(paste0("PC2 (", round(hPcv["PC2"], digits = 2), " %)")) +
  ylab(paste0("PC3 (", round(hPcv["PC3"], digits = 2), " %)")) +
  theme_bw() +
  ggtitle("Human") +
  labs(color = "Human donor", shape = "Incubation time") +
  coord_fixed(ratio = 1)

mPc2.pc3 <- mPca.df %>%
  ggplot(aes(x = PC2, y = PC3, color = animal_id, shape = timepoint)) +
  geom_point(size = 2) +
  scale_shape_manual(values = 1:2) +
  xlab(paste0("PC2 (", round(mPcv["PC2"], digits = 2), " %)")) +
  ylab(paste0("PC3 (", round(mPcv["PC3"], digits = 2), " %)")) +
  theme_bw() +
  ggtitle("Mouse") +
  labs(color = "Mouse donor", shape = "Incubation time") +
  coord_fixed(ratio = 1)
```

```r
p12 <- hPc1.pc2 + mPc1.pc2
p13 <- hPc1.pc3 + mPc1.pc3
p23 <- hPc2.pc3 + mPc2.pc3

(p12 / p13 / p23) + plot_layout(guides = "collect")
```

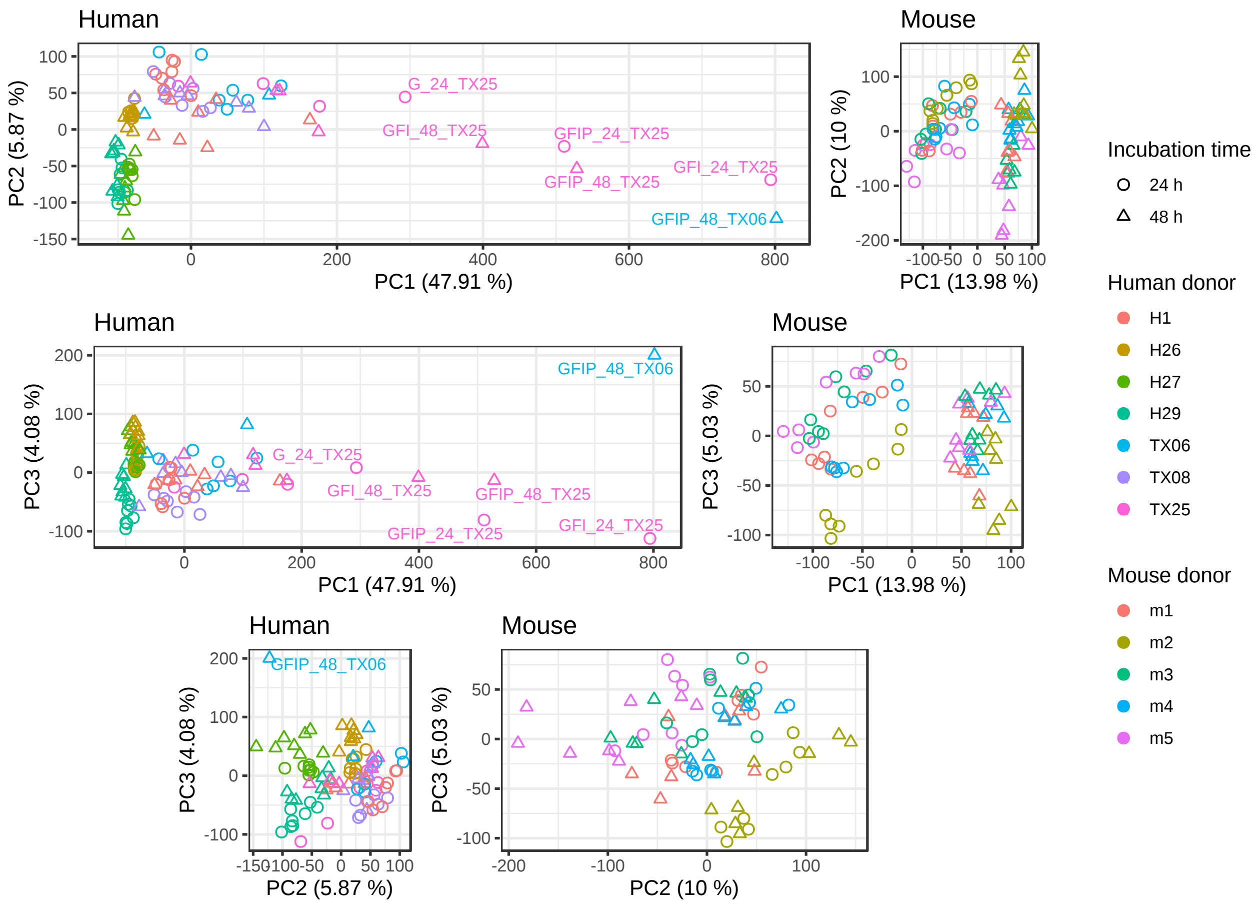


```r
ggsave("plots/Supp_Fig_S3._Principal_Component_Analysis.pdf",
       width = 10,
       height = 7)

ggsave("plots_png/Supp_Fig_S3._Principal_Component_Analysis.png",
       width = 10,
       height = 7)
```

Supplemental Figure S4. Expression variability across conditions

```
## human - compute squared coefficient of variation for each gene per group
### Initiate empty dataframe
hDf <-
  data.frame(
    ensgid = NA_character_,
    sCV = NA_integer_,
    log10mean_normCounts = NA_integer_,
    group = NA_character_
  )

### Iterate over groups
for (group in unique(sample_sheet_human$Group)) {
  ## Subset expr matrix to samples from current group and genes that have
  ## non-zero expression
  currD <-
    norm_counts_human[
      which(apply(norm_counts_human, 1, function(x) !any(x == 0))),
      sample_sheet_human %>% filter(Group == group) %>% pull(sampleId)
      ]

  ## Calculate per gene statistics within group
  currStd <- apply(currD, 1, sd)
  currMean <- apply(currD, 1, mean)
  currCV <- (currStd / currMean) ^ 2

  # sort by mean normCounts
  idx <- sort(currMean, index.return = T)$ix
  currMean <- log10(currMean[idx])
  currCV <- currCV[idx]

  currDf <-
    data.frame(
      ensgid = names(currMean),
      sCV = currCV,
      log10mean_normCounts = currMean,
      group = rep(group, length(currMean))
    )
  hDf <- rbind(hDf, currDf)
}
## remove the NA line of intialization
hDf <- hDf[-1, ]


## mouse - compute squared coefficient of variation for each gene per group
mDf <-
  data.frame(
    ensgid = NA_character_,
    sCV = NA_integer_,
    log10mean_normCounts = NA_integer_,
    group = NA_character_
  )
```

```r
for (group in unique(sample_sheet_mouse$Group)) {
  currD <-
    norm_counts_mouse[
      which(apply(norm_counts_mouse, 1, function(x) !any(x == 0))),
      sample_sheet_mouse %>% filter(Group == group) %>% pull(sampleId)
      ]

  currStd <- apply(currD, 1, sd)
  currMean <- apply(currD, 1, mean)
  currCV <- (currStd / currMean) ^ 2

  # sort by mean normCounts
  idx <- sort(currMean, index.return = T)$ix
  currMean <- log10(currMean[idx])
  currCV <- currCV[idx]

  currDf <-
    data.frame(
      ensgid = names(currMean),
      sCV = currCV,
      log10mean_normCounts = currMean,
      group = rep(group, length(currMean))
    )
  mDf <- rbind(mDf, currDf)
}
## remove the NA line of intialization
mDf <- mDf[-1, ]
```

```r
hCVplot <- ggplot(hDf,
                  aes(
                    x = log10mean_normCounts,
                    y = sCV,
                    fill = as.factor(group),
                    color = as.factor(group)
                  )) +
  geom_smooth(alpha = 0.1, linetype = 0) +
  geom_line(stat = "smooth", alpha = 0.6) +
  scale_fill_manual(values = palette_cat_16) +
  scale_color_manual(values = palette_cat_16) +
  theme_bw() +
  coord_cartesian(ylim = c(0,1)) +
  labs(fill = "Group", color = "Group") +
  xlab("log10(mean(normCounts))") +
  ylab(bquote(CV ^ {2})) +
  ggtitle("Human") +
  theme(legend.position = "none")

mCVplot <- ggplot(mDf,
                  aes(
                    x = log10mean_normCounts,
                    y = sCV,
                    fill = as.factor(group),
                    color = as.factor(group)
                  )) +
```

```
  geom_smooth(alpha = 0.1, linetype = 0) +
  geom_line(stat = "smooth", alpha = 0.6) +
  scale_fill_manual(values = palette_cat_16) +
  scale_color_manual(values = palette_cat_16) +
  theme_bw() +
  coord_cartesian(ylim = c(0,1)) +
  labs(fill = "Group", color = "Group") +
  xlab("log10(mean(normCounts))") +
  ylab(bquote(CV ^ {
    2
  })) +
  ggtitle("Mouse")

hCVplot + mCVplot + plot_layout(guides = "collect")
```

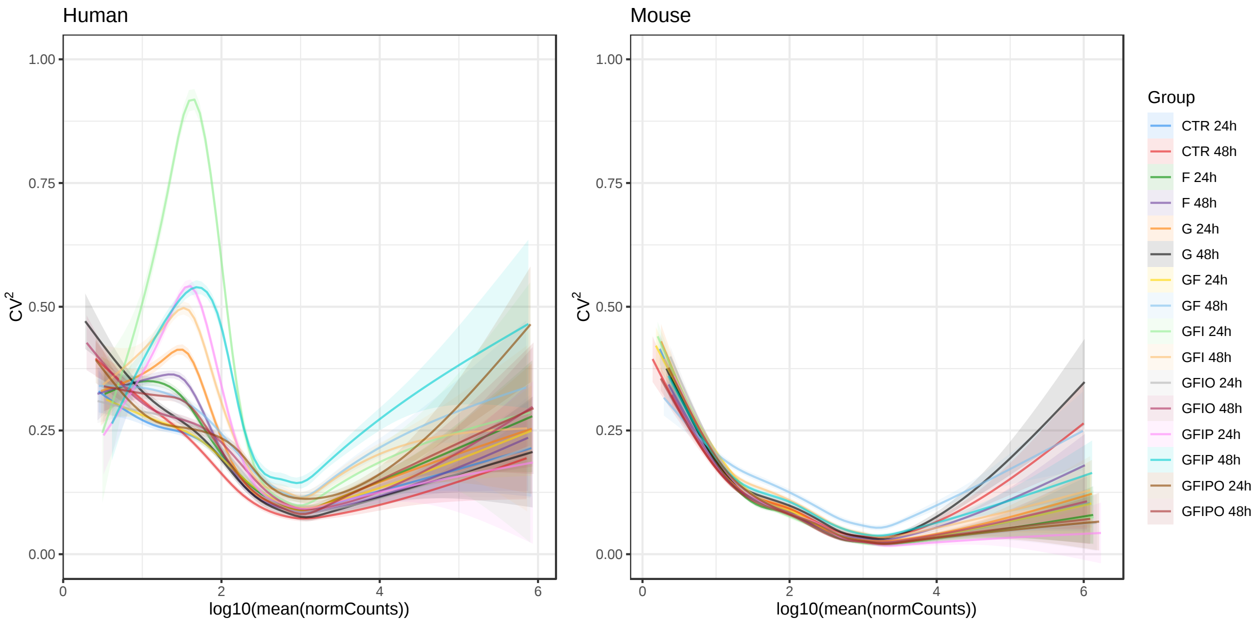


```
ggsave("plots/Supp_Fig_S4._Expression_variability_across_conditions.pdf",
       width = 7,
       height = 5)

ggsave("plots_png/Supp_Fig_S4._Expression_variability_across_conditions.png",
       width = 7,
       height = 5)
```

Supplemental Figure S5. Explained variance by Principal Component

```
## create screeplot (variance per PC)
hDf <- data.frame(PC = 1:10, var = hPcv[1:10])
p1 <- ggplot(data = hDf, aes(x = PC, y = var)) +
  geom_line(color = "grey") +
  geom_point() +
  theme_bw() +
  scale_x_continuous(limits = c(1, 10), breaks = 1:10) +
  scale_y_continuous(limits = c(0, 50), breaks = seq(0, 50, 10)) +
  ggtitle("Human") +
  ylab("% explained variance")


## create screeplot (variance per PC)
mDf <- data.frame(PC = 1:10, var = mPcv[1:10])
p2 <- ggplot(data = mDf, aes(x = PC, y = var)) +
  geom_line(color = "grey") +
  geom_point() +
  theme_bw() +
  scale_x_continuous(limits = c(1, 10), breaks = 1:10) +
  scale_y_continuous(limits = c(0, 50), breaks = seq(0, 50, 10)) +
  ggtitle("Mouse") +
  theme(axis.title.y = element_blank())

p1 + p2
```

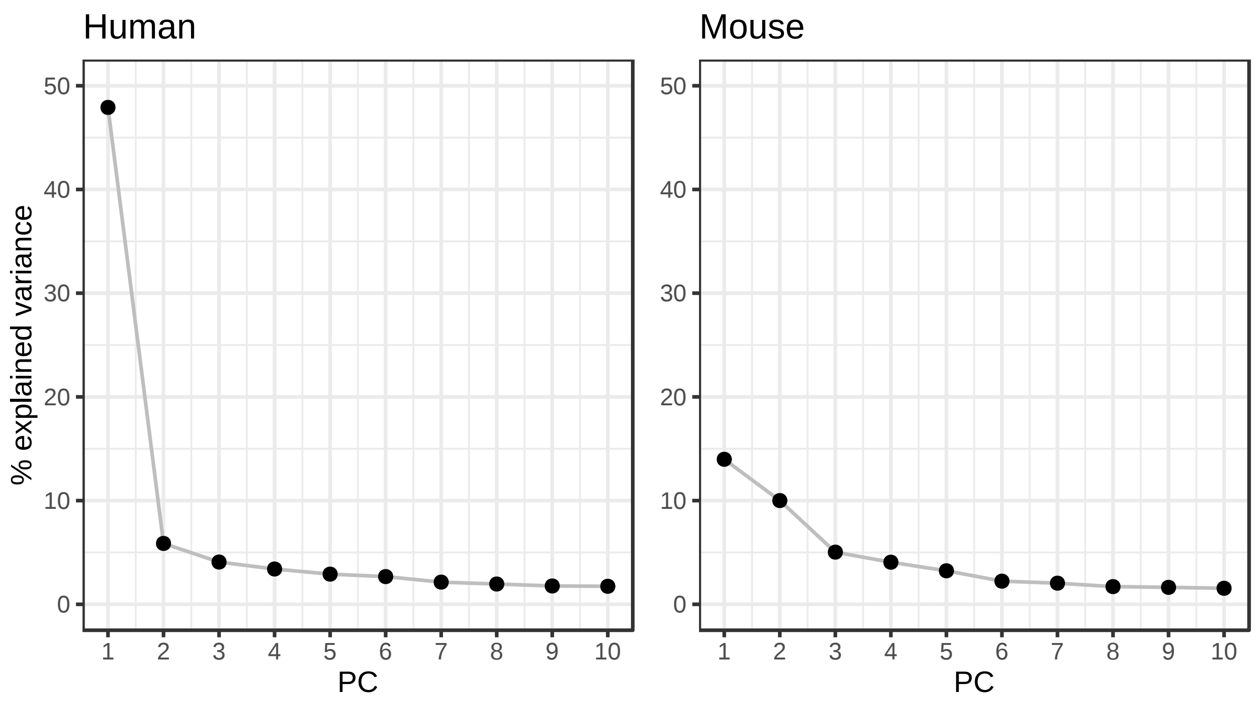


```
ggsave("plots/Supp_Fig_S5._Explained_variance_by_Principal_Component.pdf",
       width = 7,
       height = 4)
```

```
ggsave("plots_png/Supp_Fig_S5._Explained_variance_by_Principal_Component.png",
       width = 7,
       height = 4)
```

Supplemental Figure S6. Top Genes from PCA loadings

```
pc_loadings_h <- pca_human$rotation %>%
  as_tibble(rownames = "gene")

pc_loadings_m <- pca_mouse$rotation %>%
  as_tibble(rownames = "gene")


top_genes_h <- pc_loadings_h %>%
  select(ensgid = gene, PC1, PC2, PC3) %>%
  pivot_longer(starts_with("PC"), names_to = "PC", values_to = "loading") %>%
  group_by(PC) %>%
  arrange(desc(abs(loading))) %>%
  dplyr::slice(1:10) %>%
  left_join(gene_info_h %>% select(ensgid, gene_symbol), by = "ensgid")

top_loadings_h <- pc_loadings_h %>%
  inner_join(top_genes_h, by = c(gene = "ensgid"))


top_genes_m <- pc_loadings_m %>%
  select(ensgid = gene, PC1, PC2, PC3) %>%
  pivot_longer(starts_with("PC"), names_to = "PC", values_to = "loading") %>%
  group_by(PC) %>%
  arrange(desc(abs(loading))) %>%
  dplyr::slice(1:10) %>%
  left_join(gene_info_m %>% select(ensgid, gene_symbol), by = "ensgid")

top_loadings_m <- pc_loadings_m %>%
  inner_join(top_genes_m, by = c(gene = "ensgid"))
```

```
plot_pca_loadings <- function(df, x, y) {
  df %>%
    filter(PC %in% c(x, y)) %>%
    ggplot() +
    geom_hline(yintercept = 0, color = "darkblue", alpha = 0.3) +
    geom_vline(xintercept = 0, color = "darkblue", alpha = 0.3) +
    geom_segment(aes_(
      x = 0,
      y = 0,
      xend = as.name(x),
      yend = as.name(y)),
      arrow = arrow(length = unit(0.1, "in")),
      color = "brown") +
    ggrepel::geom_label_repel(
      aes_(x = as.name(x), y = as.name(y), label = ~gene_symbol),
      force = 3,
      max.time = 2,
      max.iter = 20000,
      seed = 1,
      max.overlaps = 20
    ) +
    theme_bw() +
```

```
    ylab(paste(y, "loading")) +
    xlab(paste(x, "loading"))
}
```

```
p1 <- plot_pca_loadings(top_loadings_h, x = "PC1", y = "PC2") +
    ylim(-0.004, 0.02) +
    xlim(-0.0015, 0.008) +
    ggtitle("Human")

p2 <- plot_pca_loadings(top_loadings_m, x = "PC1", y = "PC2") +
    ylim(-0.017, 0.005) +
    xlim(-0.02, 0.015) +
    ggtitle("Mouse")

p3 <- plot_pca_loadings(top_loadings_h, x = "PC1", y = "PC3") +
    ylim(-0.02, 0.01) +
    xlim(-0.02, 0.015)

p4 <- plot_pca_loadings(top_loadings_m, x = "PC1", y = "PC3") +
    ylim(-0.03, 0.022) +
    xlim(-0.015, 0.015)

p1 + p2 + p3 + p4 + plot_layout(ncol = 2)
```

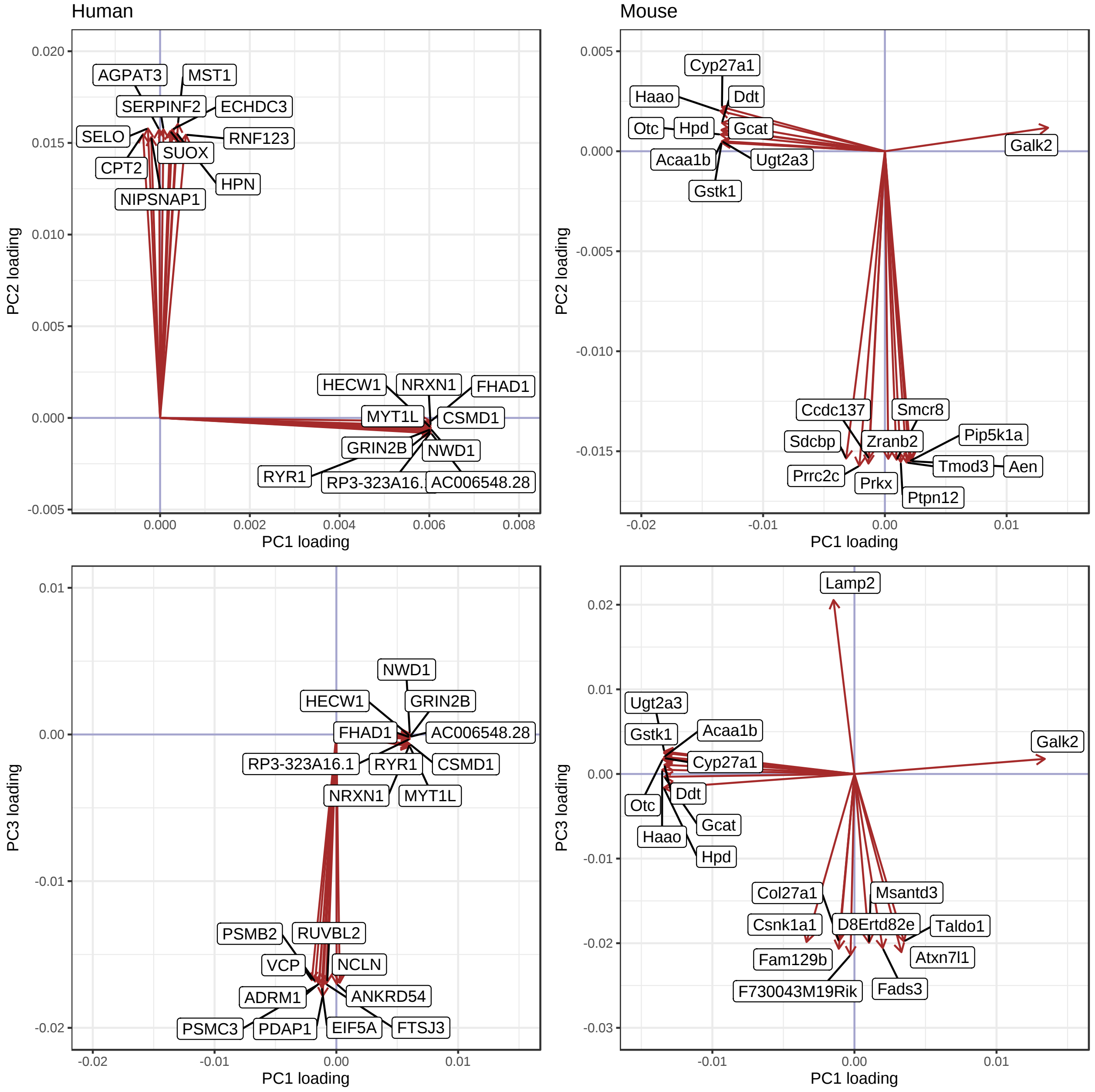


```r
ggsave("plots/Supp_Fig_S6._Top_Genes_from_PCA_loadings.pdf",
       width = 11,
       height = 11)

ggsave("plots_png/Supp_Fig_S6._Top_Genes_from_PCA_loadings.png",
       width = 11,
       height = 11)
```

Table 3. Top15 genes based on expression correlation.

```
orthologs_expressed_info <- orthologs_expressed %>%
  left_join(gene_info_h %>% select(-biotype2),
            by = c(human_ensgid = "ensgid"))

most_correlated <- orthologs_expressed_info %>%
  dplyr::slice_max(cor_pearson, n = 15)
```

```
most_correlated %>%
  mutate(cor_pearson = round(cor_pearson, 2)) %>%
  select("Gene symbol" = gene_symbol,
         "Pearson R" = cor_pearson,
         "% global identity" = global_identity_perc,
         "Type" = biotype,
         "Human ensgid" = human_ensgid,
         "Mouse ensgid" = mouse_ensgid) %>%
  gt::gt()
```

Gene symbol	Pearson R	% global identity	Type	Human ensgid	Mouse ensgid
FBN1	0.97	96	protein_coding	ENSG00000166147	ENSMUSG00000027204
MT1A	0.96	82	protein_coding	ENSG00000205362	ENSMUSG00000031765
CNNM3	0.96	86	protein_coding	ENSG00000168763	ENSMUSG00000001138
MAGED2	0.96	87	protein_coding	ENSG00000102316	ENSMUSG00000025268
STMN1	0.96	78	protein_coding	ENSG00000117632	ENSMUSG00000028832
COL6A3	0.95	74	protein_coding	ENSG00000163359	ENSMUSG00000048126
DDX56	0.95	91	protein_coding	ENSG00000136271	ENSMUSG00000004393
BICC1	0.95	92	protein_coding	ENSG00000122870	ENSMUSG00000014329
COL1A2	0.95	90	protein_coding	ENSG00000164692	ENSMUSG00000029661
MMP2	0.95	96	protein_coding	ENSG00000087245	ENSMUSG00000031740
ACKR2	0.95	72	protein_coding	ENSG00000144648	ENSMUSG00000044534
PTRF	0.94	83	protein_coding	ENSG00000177469	ENSMUSG00000004044
SMPDL3A	0.94	80	protein_coding	ENSG00000172594	ENSMUSG00000019872
COL3A1	0.94	91	protein_coding	ENSG00000168542	ENSMUSG00000026043
ERBB3	0.93	91	protein_coding	ENSG00000065361	ENSMUSG00000018166

Table 4. Top18 genes based on experimental design.

```
d0 %>%
  filter(flag_one2one == 1) %>%
  filter(abs(sum_sign) > 8) %>%
  slice_max(sum_score_h, n = 18) %>%
  select(gene_symbol, sum_score_h, ensgid, mouse_ensgid) %>%
  gt::gt()
```

gene_symbol	sum_score_h	ensgid	mouse_ensgid
FST	94.75382	ENSG00000134363	ENSMUSG00000021765
ARRDC4	67.89108	ENSG00000140450	ENSMUSG00000042659
DEPP1	63.33067	ENSG00000165507	ENSMUSG00000048489
PNPLA5	55.19282	ENSG00000100341	ENSMUSG00000018868
FADS2	39.30799	ENSG00000134824	ENSMUSG00000024665
FASN	34.90934	ENSG00000169710	ENSMUSG00000025153
ERBB3	32.17391	ENSG00000065361	ENSMUSG00000018166
YPEL3	30.92697	ENSG00000090238	ENSMUSG00000042675
PCK1	29.46294	ENSG00000124253	ENSMUSG00000027513
MID1IP1	27.98179	ENSG00000165175	ENSMUSG00000008035
LPIN2	27.15697	ENSG00000101577	ENSMUSG00000024052
ATG2A	24.60637	ENSG00000110046	ENSMUSG00000024773
ABCA9	24.30305	ENSG00000154258	ENSMUSG00000041797
OCLN	23.78411	ENSG00000197822	ENSMUSG00000021638
GRB7	23.46960	ENSG00000141738	ENSMUSG00000019312
ANGPTL8	23.43666	ENSG00000130173	ENSMUSG00000047822
HSD17B11	22.31949	ENSG00000198189	ENSMUSG00000029311
NR1I3	20.25732	ENSG00000143257	ENSMUSG00000005677

Session info

```
pander::pander(sessionInfo())
```

R version 4.1.2 (2021-11-01)

Platform: x86_64-pc-linux-gnu (64-bit)

locale: *LC_CTYPE=en_US.UTF-8*, *LC_NUMERIC=C*, *LC_TIME=en_US.UTF-8*, *LC_COLLATE=en_US.UTF-8*, *LC_MONETARY=en_US.UTF-8*, *LC_MESSAGES=en_US.UTF-8*, *LC_PAPER=en_US.UTF-8*, *LC_NAME=C*, *LC_ADDRESS=C*, *LC_TELEPHONE=C*, *LC_MEASUREMENT=en_US.UTF-8* and *LC_IDENTIFICATION=C*

attached base packages: *grid*, *stats4*, *stats*, *graphics*, *grDevices*, *utils*, *datasets*, *methods* and *base*

other attached packages: *patchwork(v.1.1.1)*, *gridExtra(v.2.3)*, *SummarizedExperiment(v.1.24.0)*, *Biobase(v.2.54.0)*, *GenomicRanges(v.1.46.1)*, *GenomeInfoDb(v.1.30.1)*, *IRanges(v.2.28.0)*, *S4Vectors(v.0.32.3)*, *BiocGenerics(v.0.40.0)*, *MatrixGenerics(v.1.6.0)*, *matrixStats(v.0.61.0)*, *ggbeeswarm(v.0.6.0)*, *forcats(v.0.5.1)*, *stringr(v.1.4.0)*, *dplyr(v.1.0.8)*, *purrr(v.0.3.4)*, *readr(v.2.1.2)*, *tidyr(v.1.2.0)*, *tibble(v.3.1.6)*, *ggplot2(v.3.3.5)* and *tidyverse(v.1.3.1)*

loaded via a namespace (and not attached): *colorspace(v.2.0-3)*, *ggsignif(v.0.6.3)*, *ellipsis(v.0.3.2)*, *XVector(v.0.34.0)*, *fs(v.1.5.2)*, *rstudioapi(v.0.13)*, *ggpubr(v.0.4.0)*, *farver(v.2.1.0)*, *ggrepel(v.0.9.1)*, *bit64(v.4.0.5)*, *fansi(v.1.0.2)*, *lubridate(v.1.8.0)*, *xml2(v.1.3.3)*, *splines(v.4.1.2)*, *ggh4x(v.0.2.1)*, *knitr(v.1.37)*, *jsonlite(v.1.8.0)*, *Cairo(v.1.5-14)*, *gt(v.0.3.1)*, *broom(v.0.7.12)*, *dbplyr(v.2.1.1)*, *compiler(v.4.1.2)*, *httr(v.1.4.2)*, *backports(v.1.4.1)*, *assertthat(v.0.2.1)*, *Matrix(v.1.4-0)*, *fastmap(v.1.1.0)*, *cli(v.3.2.0)*, *htmltools(v.0.5.2)*, *tools(v.4.1.2)*, *coda(v.0.19-4)*, *gtable(v.0.3.0)*, *glue(v.1.6.2)*, *GenomeInfoDbData(v.1.2.7)*, *tinytex(v.0.36)*, *Rcpp(v.1.0.8)*, *carData(v.3.0-5)*, *cellranger(v.1.1.0)*, *statnet.common(v.4.5.0)*, *vctrs(v.0.3.8)*, *nlme(v.3.1-155)*, *gghalves(v.0.1.1)*, *xfun(v.0.29)*, *network(v.1.17.1)*, *rvest(v.1.0.2)*, *lifecycle(v.1.0.1)*, *rstatix(v.0.7.0)*, *dendextend(v.1.15.2)*, *zlibbioc(v.1.40.0)*, *scales(v.1.2.0)*, *vroom(v.1.5.7)*, *ragg(v.1.2.1)*, *hms(v.1.1.1)*, *parallel(v.4.1.2)*, *RColorBrewer(v.1.1-2)*, *yaml(v.2.2.2)*, *pander(v.0.6.4)*, *sass(v.0.4.0)*, *yulab.utils(v.0.0.4)*, *stringi(v.1.7.6)*, *checkmate(v.2.0.0)*, *rlang(v.1.0.2)*, *pkgconfig(v.2.0.3)*, *systemfonts(v.1.0.4)*, *bitops(v.1.0-7)*, *evaluate(v.0.14)*, *lattice(v.0.20-45)*, *labeling(v.0.4.2)*, *bit(v.4.0.4)*, *tidyselect(v.1.1.1)*, *magrittr(v.2.0.2)*, *R6(v.2.5.1)*, *generics(v.0.1.2)*, *DelayedArray(v.0.20.0)*, *DBI(v.1.1.2)*, *mgcv(v.1.8-38)*, *pillar(v.1.7.0)*, *haven(v.2.4.3)*, *withr(v.2.5.0)*, *abind(v.1.4-5)*, *RCurl(v.1.98-1.6)*, *modelr(v.0.1.8)*, *crayon(v.1.5.0)*, *car(v.3.0-12)*, *utf8(v.1.2.2)*, *tzdb(v.0.2.0)*, *rmarkdown(v.2.11)*, *viridis(v.0.6.2)*, *readxl(v.1.3.1)*, *reprex(v.2.0.1)*, *digest(v.0.6.29)*, *gridGraphics(v.0.5-1)*, *textshaping(v.0.3.6)*, *munsell(v.0.5.0)*, *viridisLite(v.0.4.0)*, *beeswarm(v.0.4.0)*, *ggplotify(v.0.1.0)* and *vipor(v.0.4.5)*
